# U.S. residents’ self-reported access to veterinary care and implications for care-seeking decisions

**DOI:** 10.3389/fvets.2025.1655537

**Published:** 2025-11-06

**Authors:** Kayla Pasteur, Nicole O. Widmar, Christina V. Tran, Candace C. Croney

**Affiliations:** 1 Department of Comparative Pathobiology, Purdue University, West Lafayette, IN, United States; 2 Department of Agricultural Economics, Purdue University, West Lafayette, IN, United States; 3 School of Veterinary Medicine, Hanover College, Hanover, IN, United States; 4 Department of Animal Sciences, Purdue University, West Lafayette, IN, United States; 5 Center for Animal Welfare Science, Purdue University, West Lafayette, IN, United States

**Keywords:** Pets, owners, veterinary care-seekers, perceptions, experiences, trust in veterinarians, alternative veterinary care providers

## Abstract

**Introduction:**

Constrained access to veterinary care may significantly affect the health and welfare of millions of pets nationwide, but little is known about how pet families’ experiences with veterinary care or their perceptions of its accessibility and quality may influence their care-seeking decisions. This study examined relationships between pet owners’ demographics, perceived access to veterinary care, and related decisions and preferences.

**Methods:**

An online survey targeted 1,177 adults who were representative of the U.S. population in terms of sex, age, region, education, and income level. All participants provided their perceptions on ‘access to veterinary care’; only pet owners (*n* = 1,026) were asked about their perceptions of alternative service providers (e.g., veterinary technicians, mid-tier veterinary professionals). Similarly, only care-seekers (*n* = 236) were asked about their experiences with veterinarians; care-seekers who experienced barriers to care (*n* = 210) responded to questions regarding their willingness to seek alternative providers. Chi-square tests of independence were used to examine relationships between respondents’ demographics, perceived access to care, and preferences for veterinary service providers.

**Results:**

Of all survey participants, most defined ‘access to care’ in terms of service provider availability (78.2%), ease of communication (77.8%), and affordability (74.7%). A majority (54.7%) of care-seekers reported distrusting their veterinarians and 36.8% reported being dissatisfied with their veterinarian’s interactions with them despite being satisfied with their pet’s care. Many pet owners believed veterinary technicians (66.7%) and mid-tier veterinary professionals (65.1%) could provide quality care equivalent to a veterinarian and preferred to seek care from them rather than forego it. Age, education, and income level were related to pet owners’ perceptions of alternative providers and care-seeking decisions. Those under 45, without an advanced degree, or earning under $100,000 + reported higher levels of agreement with statements about alternative providers, indicating more favorable perceptions.

**Discussion:**

These findings illustrate how pet owners’ experiences while accessing care may influence their perceptions of veterinarians, satisfaction with them, and care-seeking decisions, including their willingness to seek alternative care providers. Further, they reiterate the need for solutions that enhance care-seeker access to high quality veterinary care and promote good animal health and welfare outcomes.

## 1 Introduction

Access to veterinary care has emerged as one of the most significant welfare crises in the United States, with major health and welfare implications for millions of companion animals across the nation (1, 2). This crisis also raises significant concerns for the well-being of those caring for pets, who may experience heightened stress when access to veterinary services is constrained (1, 3). In the last five years, dozens of scientific publications have addressed this issue (4), reflecting its growing recognition amongst major stakeholders, such as animal welfare scientists and organizations, veterinarians, and pet owners.

Several barriers documented in the scientific literature have reportedly contributed to the difficulties pet owners face when seeking timely and quality care for their animals. Among these are the rising costs of veterinary services and medications (5–7) which can create significant financial strain for pet owners, particularly those from lower-income households who may consequently choose to forego care (8, 9). Transportation is another key issue which is frequently linked to financial constraints (10). For example, difficulties accessing veterinary care are often compounded for pet owners without access to a car or who rely on public transit, as animal restrictions on public transportation limit their ability to travel to clinics (6, 11, 12). Similarly, challenges are also faced by those residing in rural communities or ‘care deserts’ where veterinary clinics may be few and far between, requiring pet owners to travel long distances to seek care (13, 14). The physical and mental health states of pet owners themselves, such as having a disability or experiencing hardship or psychological or emotional distress (e.g., due to a death in family), may further complicate their ability to seek and follow through with veterinary care (15–17). Additionally, cultural differences and language barriers can constrain access to care by impeding communication between veterinarians and their clients (18–20). Challenges related to the provision of culturally-competent care are observed in both human and animal health sectors. For example, one study found that 63% of African American patients perceived discrimination from their healthcare provider based on their race or socio-economic status, and this was correlated with reduced utilization of health services (21). Similar dynamics have emerged in veterinary care, as in some cases, clients from underserved communities have been overlooked by animal service providers because of concerns related to cost and cultural differences (6, 22). As a result, some pet owners have expressed resistance to utilizing interventions (e.g., low-cost clinics), in part because of stigmas surrounding their perceived quality of care. This has the potential to erode trust within the veterinarian-client-patient relationship (VCPR) and compromise animal health and welfare outcomes (6, 19, 21, 23, 24).

Although the volume of research on access to veterinary care is increasing, substantial knowledge gaps remain. For example, though some studies narrowly define ‘access’ in terms of service availability and affordability (25, 26), others have taken a broader approach, defining it as the availability of the economic, physical, social, mental, and emotional resources necessary to benefit from veterinary services (4). Consequently, it is still unclear how the term, ‘access to veterinary care’ is defined, perceived, and experienced by many stakeholders, including pet owners/families. The lack of a clear, widely accepted definition highlights several major concerns (4). First, it leaves room for different interpretations of what constitutes ‘access’, which can vary widely between stakeholders of different demographic backgrounds. For instance, some individuals or communities may focus on the availability and affordability of clinical services, while another may prioritize convenience of reaching a service provider, trust, or communication between service provider and client.

It is also unknown how pet owners’ perceptions of their ability to access care or the quality of veterinary care they have experienced may influence their subsequent willingness to seek care and their decision-making related to care-seeking. Evidence from one study suggests that pet owners’ expectations regarding treatment outcomes, along with the strength of the veterinarian-client relationship, can play a major role in pet owners’ decisions to utilize high-cost veterinary services and assume the associated financial burden (3). It is plausible that perceptions of access, prior experiences, and the veterinarian-client relationship may also guide future care-seeking behaviors. Insufficient knowledge about owner perceptions and quality of care experienced may contribute to confusion about what improving access to veterinary care means to the broadest group of veterinary care-seekers, which potentially complicates efforts to effectively address existing problems. Improved understanding of the interactions between all of these factors is therefore necessary to inform the development and implementation of targeted interventions that meaningfully improve both access to care and, in turn, animal welfare.

Better understanding of veterinary clients’ perceptions and experiences regarding access to pet care is also important because they (in addition to the animals for whom care is necessary) are the stakeholders most directly impacted. Greater insight in these areas, including further inquiry about barriers pet owners face and how they might relate to this population’s perceptions of veterinary care as well as their care-seeking behaviors could help inform proposed forms of client support. For example, alternative veterinary service providers, such as mid-tier veterinary health professionals, have been suggested as a possible option to bridge access gaps. However, these roles have not yet been established, the concept remains controversial in veterinary medicine (27, 28), and there is insufficient information available to gauge veterinary care-seekers’ perceptions of and willingness to utilize such providers. The objectives of this study were therefore to: (1) identify pet owners’ perceptions of access to veterinary care, their experiences seeking veterinary care, and their veterinary care preferences, (2) examine the effects of pet owner demographics on their perceptions of access to veterinary care and potential alternative care providers, and (3) understand how owners’ perceptions of access to veterinary care might influence their decision-making, including their willingness to seek care. Based on published literature, we hypothesized that some care-seekers would lack access to veterinary care, and that pet owners (particularly those encountering barriers) would hold favorable perceptions of alternative care options. We further hypothesized that pet owners’ perceived access to care, their perceptions of alternative providers, and their demographic characteristics would be related to their care-seeking decisions.

## 2 Methodology

### 2.1 Survey instrument and data collection

Qualtrics, an online survey platform (29), was used to gather information in mid-July 2024 from U.S. residents. Purdue University researchers developed, pre-tested, and designed the survey to collect the following data: (1) demographics, (2) pet ownership history, (3) perceptions of access to veterinary care and experiences seeking veterinary care, (4) perceptions of potential alternative veterinary care providers and care preferences, and (5) perceptions of animal welfare and welfare education. (Results related to item five are presented in a separate publication). With the use of quotas in Qualtrics, the sample was targeted to be representative of the U.S. population in terms of sex, age, income, education, and geographical region of residence (30–33). The regions of residence were defined according to the Census Bureau Regions and Divisions (34).

Kantar (35), a survey and data-panel management company, was used to recruit the sample of participants from their opt-in panel. Inclusion in this study required that respondents provide written consent by selecting “I agree to participate in this study” and indicate that they were 18 years of age or older. Surveys were considered completed only if respondents provided a response to at least nine of the ten demographic questions. Participants could not proceed with the remainder of the survey until they responded to these questions.

The entire survey was comprised of 37 Likert-scale, “select all that apply,” and multiple-choice questions, and was designed to be completed by participants in approximately 20 min. Utilizing Qualtrics allowed randomization of the order of statements presented in a Likert Scale format. All Likert-scale statements related to perceptions of access to veterinary care, experiences seeking veterinary care, and perceptions of potential alternative veterinary service providers were randomized except “Other,” which always appeared last in the list of statements.

**Figure 1:**
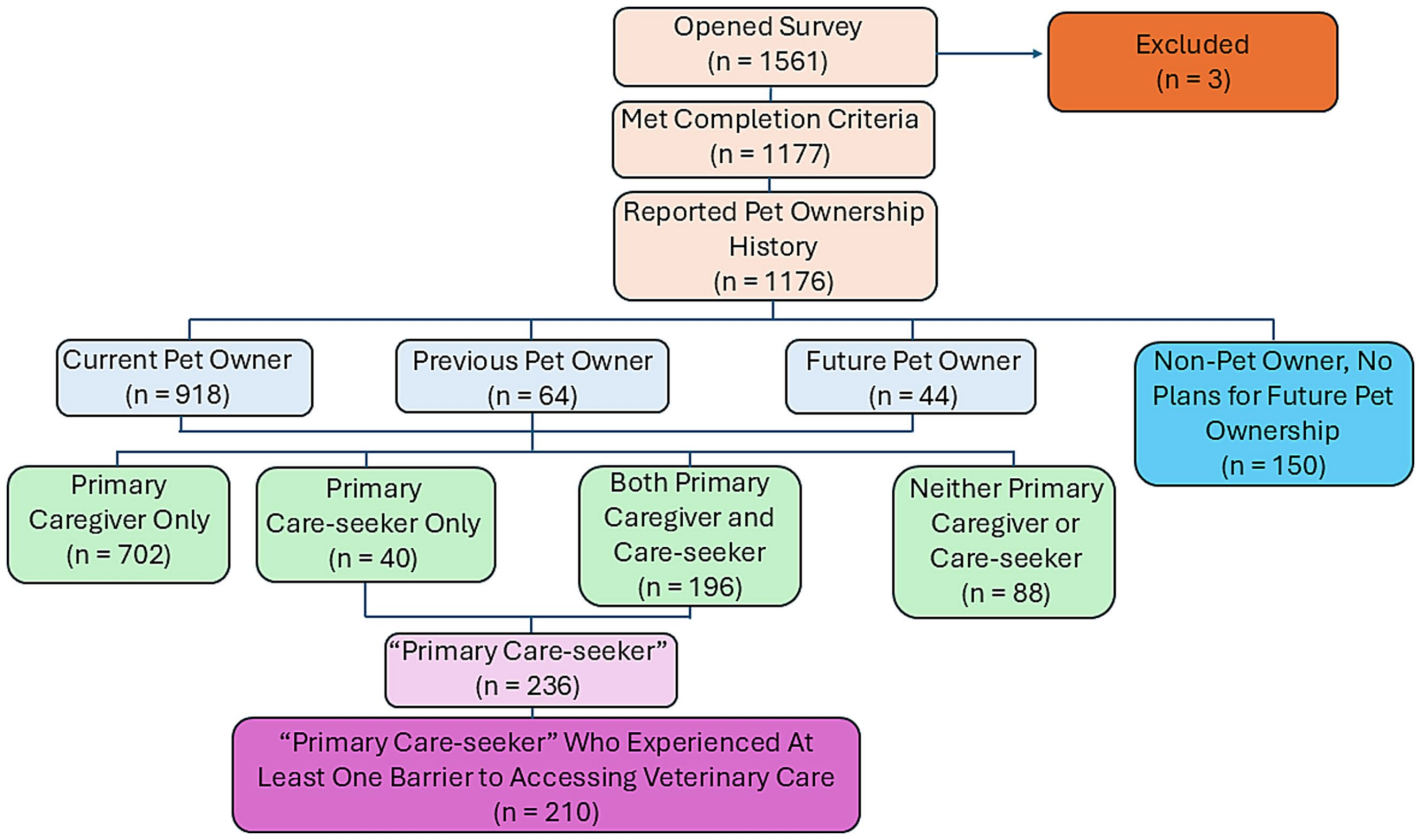
Flowchart of survey participant subsamples based on pet ownership history, role in caregiving to the animal (i.e., primary caregiver) or seeking veterinary services (i.e., primary care-seeker) for the animal, and experiences with barriers to accessing veterinary care.

To minimize survey fatigue and ensure that only relevant populations were queried in key areas, Qualtrics’ display logic was utilized to tailor some questions based on the respondents’ previous answers, resulting in several subsamples of the larger population. For instance, only those who indicated current, recent (e.g., within the last 5 years), or future (e.g., within the next 12 months) pet ownership were asked follow-up questions about their role as the primary caregiver to their animal or the primary care-seeker for veterinary services (hereafter referred to as ‘primary care-seekers’). Likewise, only those who self-identified as the primary care-seeker for their pets were asked to report the different types of veterinary care they had sought. Options included, but were not limited to, preventative care treatment and other services, such as behavioral consultations. Questions related to personal experiences seeking veterinary care were also presented only to primary care-seekers. Because the establishment of alternative veterinary care providers is currently a controversial topic amongst many stakeholders, questions relating to perceptions of these service providers were presented to all respondents who reported recent, current, or future pet ownership. Questions related to participants’ willingness to use alternative providers were shown only to care-seekers who reported experiencing at least one barrier to care, as we hypothesized that those who experienced barriers might be willing to seek alternative care options. Figure 1 illustrates the different sample populations that were created using the approaches outlined. All questions relevant to this study are provided in Supplementary materials.

### 2.2 Data analysis

Descriptive statistics were calculated for all categorical variables. Means of the five-point Likert Scale responses were analyzed, and one-way *t*-tests were used to assess the statistical significance of differences between each mean and the reported neutral values ([3], which corresponded to ‘Neutral’ on the level of agreement scale and ‘Sometimes’ on the frequency scale). The results of these analyses can be found in Supplementary Figures S1–S4. Chi-square tests of proportions were conducted to compare the demographic categories in the sample (*n* = 1,177) to that of the U.S. Census data. Relationships between respondents’ demographics (i.e., age sex, region, income level, education level), perceptions of access to veterinary care, and potential alternative veterinary care providers were examined using Chi-square tests of independence.

Preliminary analysis revealed that when using the five-point Likert scale, many cross-tabulation tables violated the assumptions of the Chi-square test of independence (see Supplementary Tables S4–S6, available in supplementary materials). Neutral responses were also ambiguous, offering limited value for interpretation. To address this, Likert scale responses for level of agreement were collapsed into a 3-point scale (e.g., [1] Disagree, [2] Neutral, [3] Agree) and neutral responses were excluded from further inferential analyses. Additionally, four demographic categories were combined (e.g., ages 18–24 and 25–34, income level 0$–$24,999 and $25,000–$49,999) to ensure that test assumptions were met. In the few cases where cross-tabulation tables still failed to meet test assumptions even after collapsing the response scales and combining demographic categories, relationships between variables were explored using the Fisher’s exact test.

In all cases where significant associations were found between variables with cross-tabulation tables larger than 2×2, the z-test of proportions was utilized in post-hoc analysis to identify the demographic sub-groups driving the association and determine significant differences between proportions. To account for the multiple comparisons that were run simultaneously, the Bonferroni correction was applied to reduce the likelihood of Type I errors. All analyses were conducted using SPSS (36), except the Fisher’s exact tests which were conducted using R version 4.5.0 (37). Statistical significance was determined using an alpha level of 0.05.

## 3 Results

### 3.1 Demographics

A total of 1,561 individuals opened the survey link which was provided to them by Kantar. Three respondents indicated that they were under 18 years old and were therefore excluded from any analysis. A total of 1,177 respondents met our inclusion criteria and completed the survey, resulting in a 75.4% completion rate. The demographic characteristics of participants who completed the survey compared to US Census proportions are presented in Table 1. The proportion of respondents who completed the survey were comparable to the US population according to the US Census (30–33) in all demographic categories except for males, those aged 65+, those who did not graduate high school, and those with an annual income of $100,000 or more. Although the proportion of males in the subsample of completed surveys differed from that of the U.S. population (*n* = 1,177, *p* = 0.02), including partially completed surveys (*n* = 1,525, *p* = 0.14) made the proportion comparable to that of U.S. Census data. Supplementary Table S1 presents comparisons between entirely completed and partially completed surveys. Table 2 shows additional demographic information, including participants’ race, ethnicity, and self-reported language proficiency.

**Table 1:** Demographic characteristics of respondents (*n* = 1,177).

Demographic variable	Respondents (*n* = 1,177) %	US census (*n* = 1,200) %
Sex		
Male^ᴪ^	43.5^*^	49.3
Female	56.5	50.7
Age		
18–24	14.4	11.4
25–34	14.9	17.5
35–44	21.6	16.9
45–54	20.1	15.7
55–64	16.6	16.5
65+	12.5^*^	22.0
Region		
Northeast	19.7	17.0
South	37.8	38.9
Midwest	20.5	20.6
West	22.0	23.6
Education level		
Did not graduate high school	3.3^*^	9.6
High school graduate, no college	26.0	29.2
Attended college, no degree earned	21.0	16.5
Attended college, bachelor’s (B.A./B.S.), associate’s, or trade degree earned	36.3	32.0
Graduate or advanced degree earned (M.S., Ph.D., Law School)	13.4	12.7
Income level		
$0–$24,999	19.5	15.8
$25,000–$49,999	23.2	18.2
$50,000–$74,999	18.9	16.2
$75,000–$99,999	15.7	12.3
$100,000+	22.8^*^	37.5

*Indicates that the proportion of respondents is statistically different than the US Census at the 0.05 level. ᴪ The proportion of males in the subsample of completed surveys is not statistically the same as the proportion of males in the U.S. population according to U.S. Census data. However, when partially completed surveys are included in the analysis, the proportion of males in the sample are statistically representative of the U.S. population according to the U.S. Census at the 0.05 level. Supplementary Table S1 presents these comparisons and is available in Supplementary material.

**Table 2:** Additional demographic information (*n* = 1,177).

Demographic variable	Respondents (*n* = 1,177) %
Race	
American Indian/Alaskan native	1.5
Asian	4.2
Black/African American	13.8
Pacific Islander/Native Hawaiian	0.3
White	75.1
Other	4.3
Prefer not to answer	0.6
Ethnicity of Latino origin	
Yes	16.1
No	83.4
Prefer not to answer	0.4
Language proficiency	
English	89.0
Spanish	1.9
English, Spanish	7.8
English, other	0.7
Other	0.3
Prefer not to answer	0.3

### 3.2 Pet ownership history

Of the 1,177 surveys that were considered completed, 1,176 respondents provided information about their pet ownership history. Most of those (78.1%) identified as current pet owners. The highest percentage of pet owners kept one (45.2%) pet, while 33.8% kept two and 21.0% kept three or more. The most frequently kept pets were dogs, with 713 respondents keeping one or more, followed by cats, which were reportedly kept by 501 respondents. Of respondents who reported pet ownership history, 5.4% reported owning a pet within the last 5 years, 3.7% planned to acquire a pet within the next 12 months, and 12.8% reported no history of pet ownership and no plans to acquire a pet.

Participants who reported being a current, recent, or future pet owner (*n* = 1,026) were asked to specify their role in the caregiving relationship with their pet(s). Most (68.4%) identified as the primary caregiver for their pet, while 3.9% identified as the primary care-seeker for veterinary services, 19.1% identified as both primary caregiver and primary veterinary care-seeker, and 8.6% reported being neither. Only those who self-identified as the primary care-seeker (*n* = 40) or as both the primary caregiver and care-seeker (*n* = 196) for their pets were presented with follow-up questions about their experiences seeking veterinary care (Figure 1).

### 3.3 U.S. residents’ perceptions of access to veterinary care

Of the full sample (*n* = 1,177) of participants who were presented with questions about how they perceived access to care, most either agreed or strongly agreed that availability of service providers (39.5% agreed, 38.7% strongly agreed), ease of communication (41.3% agreed, 36.5% strongly agreed), and affordability (37.7% agreed, 37.0% strongly agreed) are key components in defining ‘access to veterinary care’. The responses for all presented items related to what ‘access to veterinary care’ implies are available in Table 3.

**Table 3:** US residents’ self-reported perceptions of what constitutes “access to veterinary care.”

“I believe that the term ‘access to veterinary care’ implies…”	[1] Strongly disagree%	[2] Disagree%	[3] Neutral%	[4] Agree%	[5] Strongly agree%
Affordability (*n* = 1,174)	2.4	4.3	18.7	37.7	37.0
Close geographic proximity (*n* = 1,169)	1.9	3.3	21.9	40.1	32.8
Availability of service providers (*n* = 1,171)	1.7	2.7	17.4	39.5	38.7
Ease of communication (*n* = 1,171)	2.0	2.6	17.4	41.3	36.5
Disability accommodations (*n* = 1,170)	3.3	3.9	27.9	37.3	27.6
Other (*n* = 830)	6.7	3.9	47.5	21.3	20.6

These questions were presented to the full sample of 1,177 participants. The number of participants within this sample who responded to each statement are shown in the table.

### 3.4 Primary care-seekers’ experiences seeking veterinary care

**Figure 2:**
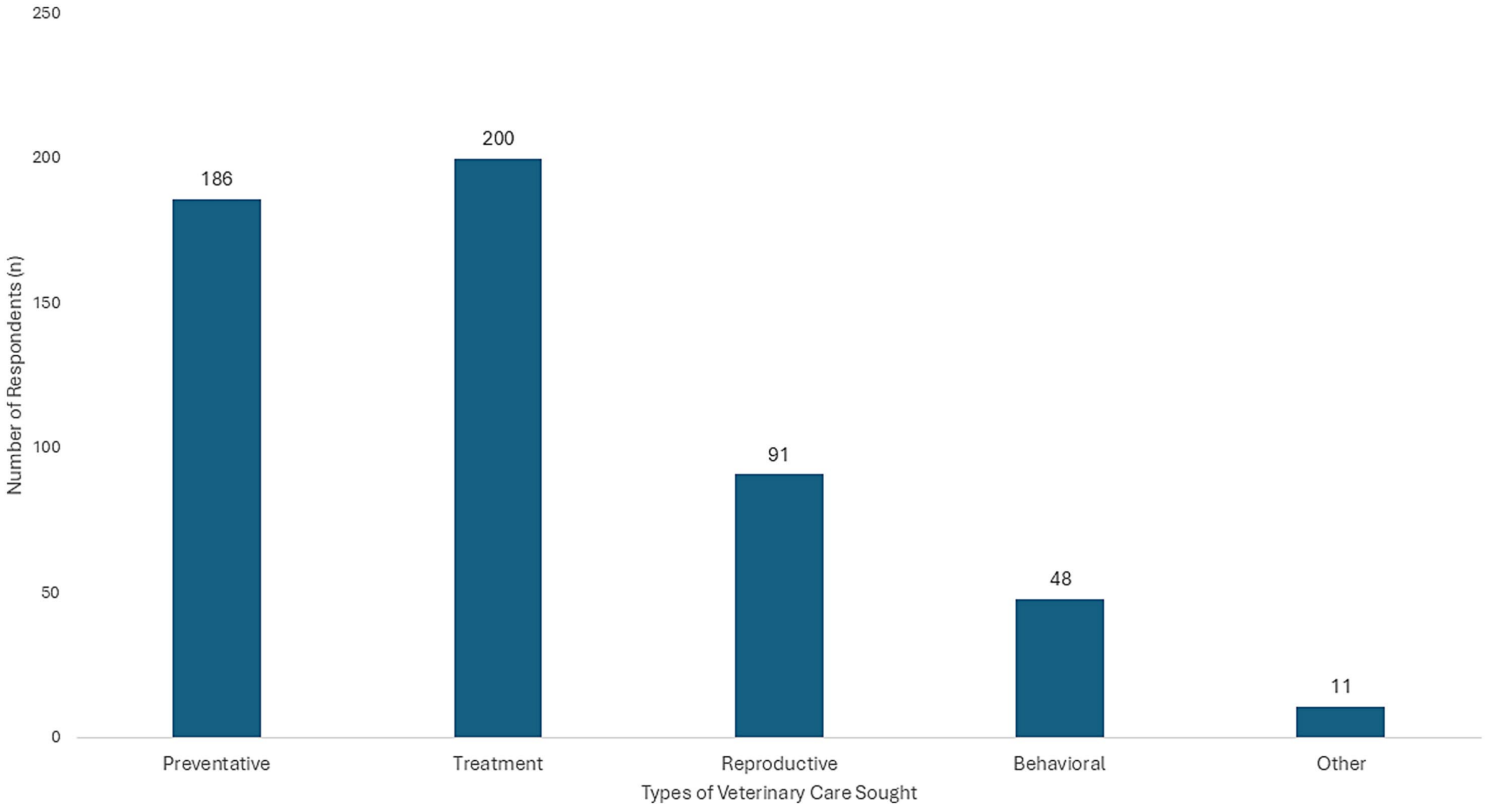
Types of veterinary care services sought by primary care-seekers (*n* = 236). In cases in which respondents indicated seeking more than one type of veterinary care, each was accounted for.

Within the subsample of respondents who self-identified as primary care-seekers (*n* = 236), the types of veterinary services sought varied and are illustrated in Figure 2. Table 4 presents the responses for all statements pertaining to primary care-seekers’ experiences seeking veterinary care. Results of preliminary analyses assessing whether mean responses significantly differed from neutral [3] are available in Supplementary Figure S1.

**Table 4:** Primary care-seekers’ self reported levels of agreement with statements relating to their experiences seeking veterinary care.

Statement	[1] Strongly disagree%	[2] Disagree%	[3] Neutral%	[4] Agree%	[5] Strongly agree%
In the area in which I live, it is easy for me to access veterinary care (*n* = 234)*	3.8	5.6	12.0	41.5	37.2
I typically see my veterinarian at least once a year (*n* = 235)*	3.0	6.8	13.2	41.3	35.7
I would prefer to have more frequent visits with my veterinarian (*n* = 236)	7.6	25.4	31.4	21.6	14.0
I would prefer to have less frequent visits with my veterinarian (*n* = 234)	7.7	24.4	30.8	26.1	11.1
It takes an unnecessarily long time to get an appointment with my veterinarian (*n* = 234)	16.7	28.2	23.9	19.7	11.5
My veterinary visits are too long (*n* = 233)	14.2	42.1	19.7	13.3	10.7
My veterinary visits are too short (*n* = 234)	9.8	36.8	23.9	17.5	12.0
During veterinary appointments, I wish I had more time with my veterinarian (*n* = 234)	6.0	26.1	29.9	23.9	14.1
My veterinarian spends an adequate amount of time with me (*n* = 234)*	3.8	26.1	12.0	41.5	37.2
I am reluctant to seek veterinary care (*n* = 234)	25.2	29.5	17.5	15.4	12.4
I have had to forego seeking veterinary care in order to meet other pressing needs (e.g., my own medical care) (*n* = 234)	20.9	27.4	16.2	24.4	11.1
Before going to a veterinarian, I would rather search online or ask a friend for a solution (*n* = 234)	10.7	24.4	23.5	26.9	14.5
I have difficulty communicating with my veterinarian (*n* = 234)	32.1	29.9	16.2	11.1	10.7
My veterinarian makes me feel comfortable when I ask questions (*n* = 234)*	2.6	3.4	15.8	44.0	34.2
My veterinarian answers questions in a way I can understand (*n* = 234)*	2.1	3.4	14.1	45.7	34.6
My veterinarian interacts with me in a way that is culturally sensitive (*n* = 234)*	2.6	7.3	27.4	38.9	23.9
I feel respected by my veterinarian (*n* = 235)*	2.1	3.0	16.6	42.1	36.2
I feel that my veterinarian really listens to my concerns. (*n* = 235)*	3.0	4.3	17.0	43.8	31.9
I believe my veterinarian possesses the necessary tools and expertise to treat my animal(s) (*n* = 235)*	2.1	3.0	10.6	47.7	36.6
I am dissatisfied with the veterinarian/veterinary care options accessible to me, but they are my only choice (*n* = 234)	29.1	33.8	16.2	14.1	6.8
I trust my veterinarian and believe they provide the best possible care (*n* = 234)	25.2	29.5	17.5	15.4	12.4

*Indicates that the mean response for the statement is statistically different than the response of 3 (Neutral) at the 0.05 level. These questions were presented to the subsample of 236 participants who identified as the primary care-seeker in their household for veterinary care. The number of participants within this sample who responded to each statement are shown in the table.

Most of the primary care-seeker subsample agreed or strongly agreed that veterinary care was easily accessible in their area of residence (41.5% agreed, 37.2% strongly agreed). Overall, 21.6% agreed and 14.0% strongly agreed that they preferred more frequent visits with their veterinarian, while slightly more reported a desire for less frequent visits (26.1% agreed, 11.1% strongly agreed). Roughly one in four primary care-seekers perceived their veterinary visits as either too long (13.3% agreed, 10.7% strongly agreed) or too short (17.5% agreed, 12.0% strongly agreed). While a vast majority reported spending an adequate amount of time with their veterinarian (41.5% agreed, 37.2% strongly agreed), over a third of primary care-seekers (23.9% agreed, 14.1% strongly agreed) expressed a desire for more time during appointments.

A large majority of primary care-seekers felt respected by their veterinarian (42.1% agreed, 36.2% strongly agreed), believed their concerns were truly heard (43.8% agreed, 31.9% strongly agreed), and reported that their veterinarian interacted with them in a culturally sensitive manner (38.9% agreed, 23.9% strongly agreed). However, more than half either disagreed (29.5%) or strongly disagreed (25.2%) with the statement, “I trust my veterinarian and believe they provide the best possible care.” About two in five primary care-seekers indicated that they would turn to online sources or friends for advice before seeking a veterinary professional (26.9% agreed, 14.5% strongly agreed) and more than a third either agreed (24.4%) or strongly agreed (11.1%) that they have foregone veterinary care altogether to prioritize other pressing needs (Table 4).

### 3.5 Primary care-seekers’ frequency of satisfaction with veterinarians

The responses to all statements relating to frequency of satisfaction with veterinarians amongst primary care-seekers (*n* = 236) are shown in Table 5. Preliminary analyses to determine whether mean responses differed significantly from sometimes [3] are shown in Supplementary Figure S2.

**Table 5:** Primary care-seekers’ frequency of self-reported satisfaction with statements relating to the quality of veterinary care and veterinarian-client interactions they experienced.

Statement	[1] Never%	[2] Rarely%	[3] Sometimes %	[4] Often%	[5] Always%
I am satisfied with both the quality of care my veterinarian provides my pet and the interactions they have with me (*n* = 236)*	1.3	5.1	13.1	37.7	42.8
I am satisfied with the quality of care my veterinarian provides my pet, but dissatisfied with the interactions they have with me (*n* = 236)*	25.4	22.9	14.8	20.3	16.5
I am dissatisfied with the quality of care my veterinarian provides my pet, but satisfied with their interactions with me (*n* = 235)*	41.3	26.0	14.5	11.1	7.2
I am dissatisfied with both the quality of care my veterinarian provides my pet and their interactions with me (*n* = 234)*	49.6	16.7	12.8	12.4	8.5

*Indicates that the mean response for the statement is statistically different than the response of 3 (Sometimes) at the 0.05 level. These questions were presented to the subsample of 236 participants who identified as the primary care-seeker in their household for veterinary care. The number of participants within this sample who responded to each statement are shown in the table.

A large majority of primary care-seekers (37.7% often, 42.8% always) were frequently satisfied with the care their pet(s) received and how their veterinarians interacted with them. However, more than a third (20.3% often, 16.5% always) reported being dissatisfied with their interactions with their veterinarian though they were often or always satisfied with the quality of care provided to their pet(s). Additionally, 12.4% of primary care-seekers were often and 8.5% were always dissatisfied with both the quality of care their pet(s) received and the interactions experienced with their veterinarian (Table 5).

### 3.6 Pet owners’ beliefs about alternative veterinary service providers and conditions under which they would prefer using their services

The subsample of participants who indicated current, previous, or future pet ownership (*n* = 1,026) was presented with questions related to their perceptions of potential alternative veterinary service providers (e.g., veterinary technicians/veterinary nurses, mid-tier veterinary professionals). These responses are shown in Table 6. In this study, a mid-tier veterinary professional was defined as an individual with more advanced education and training than a veterinary technician, similar to a nurse practitioner or physician assistant in human medicine. Supplementary Figure S3 presents the preliminary analyses of mean responses differing from neutral [3] for statements shown in Table 6.

**Table 6:** Pet owners’ self-reported levels of agreement with statements relating to perceptions of alternative veterinary service providers.

Statement	[1] Strongly disagree %	[2] Disagree %	[3] Neutral %	[4] Agree %	[5] Strongly agree %
I believe a well-trained veterinary technician/veterinary nurse can provide just as high-quality service as a veterinarian (*n* = 1,025)*	2.4	7.8	23.1	41.8	24.9
I believe a well-trained mid-tier veterinary professional can provide just as high-quality service as a veterinarian (*n* = 1,024)*	2.4	7.0	25.4	41.7	23.4
I would choose a competent veterinary technician/veterinary nurse whose demographics more closely matches mine than a veterinarian with whom there is a significant mismatch (*n* = 1,024)*	5.0	8.7	30.1	34.9	21.4
I would choose a competent mid-tier veterinary professional whose demographics more closely matches mine than a veterinarian with whom there is a significant mismatch (*n* = 1,026)*	4.2	8.4	30.6	36.3	20.6
I would choose a competent veterinary technician/veterinary nurse whose cultural sensitivity is higher than the veterinarian I currently see (*n* = 1,024)*	5.1	8.9	30.2	33.5	22.4
I would choose a competent mid-tier veterinary professional whose cultural sensitivity is higher than the veterinarian I currently see (*n* = 1,025)*	4.6	7.1	31.4	34.8	22.0

*Indicates that the mean response for the statement is statistically different than the response of 3 (Neutral) at the 0.05 level. These questions were presented to the subsample of 1,026 participants who reported current, recent, or future pet ownership. The number of participants within this sample who responded to each statement are shown in the table.

When asked about the quality of care they believed alternative veterinary service providers could offer, most pet owners either agreed or strongly agreed that a veterinary technician/veterinary nurse (41.8% agreed, 24.9% strongly agreed) or mid-tier veterinary professional (41.7% agreed, 23.4% strongly agreed) could provide just as high-quality care as a veterinarian. A majority of pet owners also agreed or strongly agreed that they would choose a competent veterinary technician/veterinary nurse under conditions where the veterinary technician/veterinary nurse’s demographics aligned better with their own (34.9% agreed, 21.4% strongly agreed), and if their cultural sensitivity was higher than that of their current veterinarian (33.5% agreed, 22.4% strongly agreed). Similar results were found for mid-tier veterinary professionals (Table 6).

### 3.7 Willingness and preference to utilize alternative veterinary service providers among primary care-seekers experiencing barriers to care

Table 7 presents responses of care-seekers who experienced at least one barrier to accessing care (*n* = 210) to statements related to their willingness to seek alternative veterinary service providers. Preliminary analyses determining whether mean responses to the statements in Table 7 significantly differed from neutral [3] are provided in Supplementary Figure S4. Of the 210 care-seekers who reported experiencing at least one barrier to accessing veterinary care, most agreed or strongly agreed that they would be willing to seek care from a veterinary technician/veterinary nurse (41.4% agreed, 24.3% strongly agreed) or mid-tier veterinary professional (39.5% agreed, 22.4% strongly agreed) in circumstances where they encountered barriers to accessing a veterinarian. A majority also reported that they would prefer to seek care from a veterinary technician/veterinary nurse (41.1% agreed, 24.9% strongly agreed) or mid-tier veterinary professional (38.3% agreed, 23.0% strongly agreed) rather than forego care (Table 7).

**Table 7:** Primary care-seekers’ self-reported levels of agreement with statements relating to willingness to seek alternative veterinary service providers care in circumstances where barriers to care are encountered.

Statement	[1] Strongly Disagree %	[2] Disagree %	[3] Neutral %	[4] Agree %	[5] Strongly agree %
In circumstances where I experience barriers to accessing a veterinarian, I would be willing to see a veterinary technician/veterinary nurse (*n* = 210)*	4.3	9.0	21.0	41.4	24.3
In circumstances where I experience barriers to accessing a veterinarian, I would be willing to see a mid-tier veterinary professional (*n* = 210)*	4.3	5.7	28.1	39.5	22.4
In circumstances where I experience barriers to accessing a veterinarian, I would prefer to see a veterinary technician/veterinary nurse rather than forego care (*n* = 209)*	3.3	6.7	23.9	41.1	24.9
In circumstances where I experience barriers to accessing a veterinarian, I would prefer to see a mid-tier veterinary professional rather than forego care (*n* = 209)*	2.4	11.0	25.4	38.3	23.0

*Indicates that the mean response for the statement is statistically different than the response of 3 (Neutral) at the 0.05 level. These questions were presented to the subsample of 210 participants who identified as the primary care-seeker for veterinary care and reported experiencing at least one barrier to accessing veterinary care. The number of participants within this sample who responded to each statement are shown in the table.

### 3.8 Relationships between pet owners’ demographics, beliefs about, and conditions under which they would prefer alternative veterinary care providers

Several associations were found between pet owners’ demographics and their perceptions of potential alternative care providers, with relationships being most frequently observed between age and income levels. Results from all Chi-square tests of independence involving demographic variables are presented in Supplementary Table S2, with corresponding *post hoc* analyses shown in Tables 8, 9.

**Table 8:** Relationships between participants’ demographics (sex, age, region) and their self-reported with statements relating to perceptions of alternative veterinary service providers.

Statement		Sex		Age				Region			
		Male (%)	Female (%)	18–34 (%)	35–44 (%)	45–54 (%)	55–65 + (%)	Northeast (%)	South (%)	Midwest (%)	West (%)
I believe a well-trained veterinary technician/veterinary nurse can provide just as high-quality service as a veterinarian (*n* = 788)	[3] Agree	84.2	88.5	92.2_a_	91.0_a_	86.5_a, b_	75.3_b_	87.5	86.4	84.8	88.0
	[1] Disagree	15.8	11.5	7.8_a_	9.0_a_	13.5_a, b_	24.7_b_	12.5	13.6	15.2	12.0
I believe a well-trained mid-tier veterinary professional can provide just as high-quality service as a veterinarian (*n* = 764)	[3] Agree	87.2	87.4	88.8	90.9	87.2	81.7	88.1	86.5	85.6	89.5
	[1] Disagree	12.8	12.6	11.2	9.1	12.8	18.3	11.9	13.5	14.4	10.5
I would choose a competent veterinary technician/veterinary nurse whose demographics more closely matches mine than a veterinarian with whom there is a significant mismatch (*n* = 716)	[3] Agree	80.4	80.5	83.5_a, b_	86.1_a_	75.5_a, b_	74.4_b_	79.6	80.0	78.6	83.5
	[1] Disagree	19.6	19.5	16.5_a, b_	13.9_a_	24.5_a, b_	25.6_b_	20.4	20.0	21.4	16.5
I would choose a competent mid-tier veterinary professional whose demographics more closely matches mine than a veterinarian with whom there is a significant mismatch (*n* = 712)	[3] Agree	82.0	81.8	87.1	83.1	76.5	77.7	83.6	81.6	78.9	83.5
	[1] Disagree	18.0	18.2	12.9	16.9	23.5	22.3	16.4	18.4	21.1	16.5
I would choose a competent veterinary technician/veterinary nurse whose cultural sensitivity is higher than the veterinarian I currently see (*n* = 715)	[3] Agree	80.6	79.6	84.8_a, b_	90.1_b_	75.9_a,c_	65.8_c_	77.8	80.8	74.8	84.9
	[1] Disagree	19.4	20.4	15.2_a, b_	9.9_b_	24.1_a,c_	34.2_c_	22.2	19.2	25.2	15.1
I would choose a competent mid-tier veterinary professional whose cultural sensitivity is higher than the veterinarian I currently see (*n* = 703)	[3] Agree	82.3	83.4	87.7_a_	88.6_a_	81.5_a, b_	71.2_b_	82.2	82.5	79.9	86.9
	[1] Disagree	17.7	16.6	12.3_a_	11.4_a_	18.5_a, b_	28.8_b_	17.8	17.5	20.1	13.1

Demographic questions were presented to the full sample of 1,177 participants. Questions relating to perceptions of alternative veterinary service providers were presented to the subsample of 1,026 participants who reported current, recent, or future pet ownership. The number of participants within this sample who responded to the questions with agreement (Agree, Strongly Agree) or disagreement (Disagree, Strongly Disagree) are shown in the table. As determined by the z-test of proportions (for tables larger than 2×2), statistically significant differences between columns within each demographic category (sex, age, region) at the 0.05 level are denoted by different letter subscripts (a, b). Within a given row, values that share the same subscript do not statistically differ, while values with different subscripts do. In cases where there are no subscripts shown, no significant associations were observed for post-hoc comparisons. For example, when reading the “Agree” row, there were no associations found between sex and the first statement shown resulting in no subscripts in either the “Male” or “Female” column. In that same row, there were associations found between age and the first statement shown, with subscripts a and b indicating significant differences between the 18–34 and 55–65 + columns.

**Table 9:** Relationships between participants’ demographics (education level, income level) and their levels of agreement with statements relating to perceptions of alternative veterinary service providers.

Statement		Income level				Education level			
		0$–$49,999 (%)	$50, 000–$74,999 (%)	$75,000–$99,999 (%)	$100,00 + (%)	High school graduate or less (%)	Attended college, no degree earned (%)	Attended college, bachelor’s (B.A./B.S.), associate’s, or trade degree earned (%)	Graduate or advanced degree earned (M.S., Ph.D., Law School) (%)
I believe a well-trained veterinary technician/veterinary nurse can provide just as high-quality service as a veterinarian (*n* = 788)	[3] Agree	87.6	89.4	90.1	79.8	87.8	89.0	86.3	80.9
	[1] Disagree	12.4	10.6	9.9	20.2	12.2	11.0	13.7	19.1
I believe a well-trained mid-tier veterinary professional can provide just as high-quality service as a veterinarian (*n* = 764)	[3] Agree	86.9	90.1	91.7	81.9	87.1	85.4	89.9	83.5
	[1] Disagree	13.1	9.9	8.3	18.1	12.9	14.6	10.1	16.5
I would choose a competent veterinary technician/veterinary nurse whose demographics more closely matches mine than a veterinarian with whom there is a significant mismatch (*n* = 716)	[3] Agree	83.8_a_	79.7_a, b_	86.0_a_	71.7_b_	81.4_a, b_	81.1_a, b_	83.0_a_	69.2_b_
	[1] Disagree	16.2_a_	20.3_a, b_	14.0_a_	28.3_b_	18.6_a, b_	18.9_a, b_	17.0_a_	30.8_b_
I would choose a competent mid-tier veterinary professional whose demographics more closely matches mine than a veterinarian with whom there is a significant mismatch (*n* = 712)	[3] Agree	87.2_a_	79.1_a, b_	85.6_a_	72.5_b_	84.4_a_	79.5 _a, b_	84.6_a_	70.1_b_
	[1] Disagree	12.8_a_	20.9_a, b_	14.4_a_	27.5_b_	15.6_a_	20.5 _a, b_	15.4_a_	29.9_b_
I would choose a competent veterinary technician/veterinary nurse whose cultural sensitivity is higher than the veterinarian I currently see (*n* = 715)	[3] Agree	83.4_a_	79.0_a, b_	87.6_a_	68.9_b_	82.0	83.2	79.9	70.5
	[1] Disagree	16.6_a_	21.0_a, b_	12.4_a_	31.1_b_	18.0	16.8	20.1	29.5
I would choose a competent mid-tier veterinary professional whose cultural sensitivity is higher than the veterinarian I currently see (*n* = 703)	[3] Agree	85.1_a, b_	78.7_a_	93.1_b_	75.0_a_	83.8	81.0	85.7	75.8
	[1] Disagree	14.9_a, b_	21.3_a_	6.9_b_	25.0_a_	16.2	19.0	14.3	24.2

Demographic questions were presented to the full sample of 1,177 participants. Questions relating to perceptions of alternative veterinary service providers were presented to the subsample of 1,026 participants who reported current, recent, or future pet ownership. The number of participants within this sample who responded to the questions with agreement (Agree, Strongly Agree) or disagreement (Disagree, Strongly Disagree) are shown in the table. As determined by the z-test of proportions (for tables larger than 2×2), statistically significant differences between columns within each demographic category (education, income) at the 0.05 level are denoted by different letter subscripts (a, b). Within a given row, values that share the same subscript do not statistically differ, while values with different subscripts do. In cases where there are no subscripts shown, no significant associations were observed for post-hoc comparisons. For example, when reading the “Agree” row, there were no associations found between education level and the fifth statement shown resulting in no subscripts in any columns representing education levels. In that same row, there were associations found between income level and the fifth statement shown, with subscripts a and b indicating significant differences between the $0–$49,999 and $100,000 + columns.

### 3.9 Relationships between primary care-seekers’ demographics, perceptions of access to veterinary care, and beliefs about alternative veterinary service providers

Primary care-seekers’ (*n* = 236) responses to the statement, “In the area in which I live, it is easy for me to access veterinary care” were associated with region (Χ^2^_3_ = 6.39, *p* = 0.094, Fisher’s exact test *p* = 0.044). However, post-hoc analysis revealed no differences between the proportions of regional demographic subgroups (e.g., northeast, south). There were no relationships observed between care-seekers’ perceived access to care and their responses to statements related to perceptions of alternative veterinary service providers. The results from all Chi-square tests of independence involving respondents’ perceived access to care are shown in Supplementary Table S3.

### 3.10 Relationships between demographics, perceptions of access to care, and preferences for and willingness to utilize alternative veterinary service providers among care-seekers experiencing barriers

No associations were found between the demographics of the 210 care-seekers who reported experiencing one or more barriers to care and their willingness to seek care from either a veterinary technician/veterinary nurse or mid-tier veterinary professional. However, a relationship was observed between primary care-seeker age and preference to seek care from a veterinary technician/veterinary nurse rather than forego it (Χ^2^_3_ = 11.41, *p* = 0.01). Of those who would prefer to seek care from a veterinary technician or nurse, fewer participants were aged 45–54 (15.9%) compared to those aged 18–34 (30.4%).

Care-seekers’ perceived access to care was associated with their willingness to seek care from a veterinary technician/veterinary nurse where barriers to accessing a veterinarian (Χ^2^_1,_ = 5.08, *p* = 0.024, Fisher’s exact test *p* = 0.036) were experienced. Of those who reported easy access to care, a majority of respondents (85.1%) agreed they would be willing to seek veterinary technicians/nurses as an alternative. Likewise, most of those without access to care agreed (62.5%) that they would be willing to seek care from a veterinary technician.

Similar associations were observed between care-seekers’ perceived access to care and their responses to the statements “In circumstances where I experience barriers to accessing a veterinarian, I would prefer to see a veterinary technician/veterinary nurse rather than forego care” (Χ^2^_1,_ = 5.79, *p* = 0.016, Fisher’s exact test *p* = 0.031) and “In circumstances where I experience barriers to accessing a veterinarian, I would prefer to see a mid-tier veterinary professional rather than forego care” (Χ^2^_1,_ = 7.24, *p* = 0.007, Fisher’s exact test p = 0.016). Among respondents with easy access to veterinary care, a majority indicated a preference to seek care from either a veterinary technician (89.8%) or mid-tier veterinary professional (85.7%) if the alternative was to forego care altogether. Of those who reported not having access to care, many also agreed that they would prefer to seek care from a veterinary technician (68.8%) or mid-tier veterinary professional (57.1%) as an alternative.

## 4 Discussion

This study aimed to understand U.S. pet owners’ perceptions of access to veterinary care, their experiences and preferences in seeking care, and examine relationships between these, demographics factors and decision-making relative to care-seeking behavior. Our hypotheses that these factors might interact with each other and demographic variables to influence care-seeking decisions were largely supported, although some of the results were unexpected.

An interesting finding was the large discrepancy between the number of participants who identified as the primary caregiver of their pet (68.4%) (i.e., being responsible for their feeding, exercise and daily care) and those identifying as the primary care-seeker (3.9%) for veterinary services or as the primary for both (19.1%). This discovery highlights the importance of delineating between these sub-populations as doing so offers the opportunity to gauge whether and to what degree responses to surveys about perceptions of veterinary care and access to it are based on direct experience, and are more likely to accurately represent the experiences of the broader population of care-seekers. Studies that overlook this distinction may risk under-representing the perspectives of those with the greatest level of direct experience in trying to access veterinary care in favor of representing the broader category of pet owners’ perceptions, some of which may not be as relevant to the questions of interest. This may result in data sets and conclusions that do not optimally inform efforts to improve access to veterinary care.

In regard to conceptions of ‘access to veterinary care’, the finding that respondents most frequently identified service availability (78.1%), ease of communication (77.9%), and affordability (74.7%) as key components in defining access mirrors what is observed in the human healthcare literature (38, 39), where some definitions also include service availability, affordability, utilization, as well as social and cultural barriers (38). Clear understanding of veterinary-client definitions of access is essential for identifying and addressing potential gaps and solutions needed by pet-keeping households.

Our hypothesis that a large proportion of care-seekers would report lack of access to veterinary care was not met. Similar to the findings of King et al. (5), a large majority of care-seekers in this study agreed that veterinary care was accessible in their communities. However, this conflicts with previous studies (1, 6, 40). Our results may differ from previous findings because our study was specifically designed to identify the perceptions of individuals with experience seeking veterinary care, whereas other studies may reflect the experiences of both care-seekers and non-care-seekers. Another possible explanation is that care-seekers’ responses may have reflected some degree of social desirability bias, as a desire to appear more capable of accessing and providing veterinary care for their pets could have deterred some from reporting challenges they experienced (41). This suggestion is supported by our finding that for each barrier listed, 22.1% or more of care-seekers indicated they had experienced it, despite most respondents indicating good access to care. Additionally, 35.5% reported foregoing care to prioritize more urgent needs, such as their own medical care.

Although surprising, no relationships were observed between care-seekers’ perceived access to care and any demographic factors. This outcome is likely due to the small subsample size which successfully targeted the most relevant participants, but may have limited our ability to detect differences between demographic groups. Future studies could mitigate this limitation by recruiting through multiple channels or extending the survey’s administration period to ensure a larger and more representative sample. Alternatively, the sub-population of interest (e.g., pet owners, primary care-seekers, or care-seekers who experience barriers) could be targeted at the onset of future studies.

Notably, over 20% of respondents experienced difficulties in getting an appointment and indicated a desire for more time with their veterinarian. More than a third reported being frequently dissatisfied with the interactions they had with their veterinarian despite being satisfied with the care their pet received. Identifying that one in five care-seekers face these challenges in accessing quality veterinary care highlights the need for further investigation into whether such difficulties are concentrated in specific regions of the United States or disproportionately affect certain demographic groups. As there are direct implications for animal welfare if clients cannot access veterinary care, and human well-being (e.g., excessive stress) as noted by Applebaum et al. (15), future studies should explore these patterns to better inform interventions targeted to improve accessibility and equity in veterinary care.

Moreover, our results highlight that ‘access’ extends beyond appointment availability and must also take into account the quality of care received by the client. This includes giving the client the time they need. Although most care-seekers reported spending an adequate amount of time with their veterinarian, many desired even more time, highlighting a need for increased veterinarian-client interaction and reiterating the importance of addressing gaps in access to care. It is plausible that this finding may be connected to some of the dissatisfaction with their veterinarians respondents reported. In the human healthcare literature younger patients’ satisfaction levels were found to be influenced by their perceptions that the physician was caring and did not rush the doctor-patient interaction (42). Similarly, in veterinary care, if clients feel that they are paying for care but not getting sufficient time with their veterinarian, their perceptions of the value of the service received, their trust in the veterinary-client relationship, and willingness to engage in future care-seeking behaviors may all be negatively impacted.

In exploring respondents’ experiences with care-seeking, our study revealed a surprising finding that more than half of care-seekers (54.7%) reported distrust in their veterinarian, which highlights a critical issue for the veterinary community. Because previous studies suggest that the public perceives veterinarians more favorably than other medical professionals [e.g., human physicians (43)] and veterinarians are among the most trusted sources of animal welfare information (44), our findings were unexpected. The lack of trust observed in this sample may be due to several factors which require further investigation. First, sampling only primary care-seekers may have allowed us to uncover insights that are obscured when those who never or only occasionally seek care are included in the data, especially if those individuals have a more positive overall perception. It is also probable that being unable to get an appointment without long wait times, then experiencing visits that appear too short, in combination with negative previous experiences, ineffective communication, and rising care costs serves to undermine trust. Future studies should explore these concepts to determine if trust in veterinarians may differ between care-seeker and non-care-seeker populations and whether it varies depending on clients’ previous experiences. Distrust in veterinarians among care-seekers poses a threat to animal health and welfare, impacting service utilization, treatment compliance, and client retention.

Relatedly, it is important to note that nearly half of respondents indicated a preference for seeking solutions regarding animal care from peers or online resources before seeing a veterinary professional. This finding may reflect a degree of distrust, especially for respondents who did not believe their veterinarian provided the best possible care. It is possible that it also indicates a desire to avoid veterinary costs unnecessarily or to ensure that time and money expended on veterinary visits are “well spent” or both. This reiterates the importance of bridging trust gaps and considering affordability and value of services as components of improved access to care, as seeking veterinary care solutions from inappropriate sources puts animal health and welfare at risk. Additional research should investigate whether care-seekers’ preference for obtaining animal care advice from peers or online sources is related to perceived convenience, the value of services, trust—or lack of trust—in their veterinarian, or a combination of these factors.

Among the most intriguing of our findings were pet owners’ perceptions of potential alternative veterinary care providers and care-seekers’ willingness to utilize these alternatives. We hypothesized that pet owners and care-seekers, particularly those who experienced difficulties accessing a veterinarian, would respond favorably to alternative care options. This hypothesis was somewhat met as many respondents reported positive perceptions of potential alternative veterinary care providers and expressed their willingness and preference to seek care from professionals other than veterinarians. The mid-tier veterinary professional role, often referred to as a Veterinary Professional Associate (VPA) has been the subject of contentious debate within the animal health and welfare communities (28, 45–47) and other major stakeholders. Proponents of this model highlight potential benefits, including increased access to care, improved care efficiency, and cost-effectiveness (48, 49), ensuring more pets receive necessary health services without overwhelming the existing veterinary workforce. However, opponents argue that the risks associated with such a role may outweigh the benefits, highlighting challenges related to appropriate educational preparation, student debt-to-income ratio, regulation, licensing frameworks, and potential confusion regarding the responsibilities of this role (28, 50). Such confusion could further undermine trust in the veterinary healthcare system, potentially leading pet owners to seek care from inappropriate sources, or avoid seeking care altogether.

In apparent contrast to our results, a survey conducted by the AVMA found that 79% of pet owners wanted a licensed veterinarian in charge of their pet’s care, and 80% believed it would be dangerous for any alternative care provider to make healthcare decisions for their pet (51). However, our findings suggest that a large majority of pet owners believe a well-trained veterinary technician/veterinary nurse (66.7%) or mid-tier veterinary professional (65.1%) could provide service of equal quality to a veterinarian. Most care-seekers who experienced barriers to veterinary care (e.g., cost of veterinary services or distance to a service provider) expressed both a willingness and preference to seek care from a potential alternative provider rather than forego care, highlighting the influence that barriers to care may have on care-seeking decisions. These findings closely align with those of Niemiec et al. (47), who found that most pet owners were comfortable with certified veterinary technicians (CVTs) and mid-level practitioners or veterinary professional associates (VPAs) performing treatment of non-urgent medical conditions (52.1, 49.7%), annual examinations (48.6, 41.3%), and vaccine administration (58.1, 46.7%), respectively.

Our hypothesis that relationships would be found between perceived access to care and willingness to seek alternative service providers was met. However, the types of relationships observed were somewhat unexpected. Among respondents with good access to care, a larger proportion preferred to seek care from a mid-tier veterinary professional rather than forego care. While this finding was surprising, it may suggest that even those with access to a veterinarian value having additional care options. This may be especially true if they perceive mid-tier veterinary professionals as more affordable or convenient. Similarly, of those who did not report good access to care, a majority (57.1%) preferred seeking a mid-tier veterinary professional to foregoing care altogether. It should be noted, however, that 42.9% of care-seekers with constrained access to a veterinarian disagreed that they would choose seeking an alternative provider over foregoing care. This finding suggests that interventions aimed at offering services from individuals other than veterinarians may not appeal to all care-seekers who lack access. These findings also demonstrate that there is substantial interest in alternative service providers extending beyond Colorado and the western United States to a national sample, particularly among care-seekers with access to a veterinarian. Whether those in our study truly understand the potential animal health and welfare implications of choosing a care provider who is not a veterinarian remains unclear, however.

A majority of pet owners indicated a preference for a competent veterinary technician/veterinary nurse or mid-tier veterinary professional who demonstrated greater cultural sensitivity than their current veterinarian, and whose demographics more closely aligned with their own. This finding underscores the high priority many U.S. pet owners place on accessing culturally competent care. Agreement with related statements was frequently associated with demographics, with younger participants (under 45), those without a graduate or advanced degree, and those earning under $100,000 annually reporting higher levels of agreement. This aligns with reports of growing demand for culturally competent care (52) and suggests that pet owners within these demographic groups may be more likely to seek alternative providers when these needs are unmet. In this regard, it is possible that veterinary clients may be responding similarly to human patients surveyed, whose responses indicate that those with higher levels of education often feel more respected by their physicians compared to those with less education (53). Many human patients also report being treated unfairly or with disrespect by their doctors due to their race, ethnicity, or ability to speak English (53). The degree to which cultural competence and demographic identification with one’s veterinarian might also influence trust and the veterinarian-client relationship should be further examined (52, 54). However, it should be noted that in our study, 62.8% of *care-seekers* reported culturally sensitive interactions with their veterinarian, suggesting that veterinarians are generally effective in providing culturally competent care. Given the importance of cultural competence to pet owners and the finding that a minority (9.9%) of care-seekers did not experience culturally sensitive interactions with their veterinarians, future research should examine whether primary care-seekers’ level of trust in veterinarians is influenced by shared demographic characteristics or perceived cultural competency, and how these relationships may differ across demographic groups.

### 4.1 Limitations

A limitation of this study, as noted previously, is that some demographic subgroups within our sample, were not proportionally representative of the U.S. population according to U.S. Census data (30–32). These included male participants, those aged 65 and older, those who did not graduate high school, and those who earned an annual income of $100,000 or more. This underrepresentation may be attributed to several factors. Males are often less likely to participate in online surveys and have lower response rates compared to female participants, possibly due to perceived lack of interest or time constraints (55–57). Older adults were also underrepresented in this study, possibly due to technological barriers or a lack of interest in online survey participation (58, 59). Individuals without a high school diploma may have been underrepresented due to time or occupational constraints, as they are more likely to refuse participation than those with higher levels of education (60). When specific demographic groups are underrepresented, the survey results may not fully reflect the perceptions of the broader population and therefore must be interpreted with caution.

Another limitation pertains to the design of the Likert scale questions related to the perceptions of alternative veterinary service providers. The research team intentionally provided only a very brief description of ‘mid-tier veterinary professional’ and did not differentiate between the services offered by veterinary technicians/veterinary nurses and those that would be offered by mid-tier veterinary professionals. However, we did provide participants with some context, comparing mid-tier veterinary professionals to nurse practitioners or physician assistants in the human medical field. While these comparisons are frequently used (48, 61), it might have been helpful to outline the specific training and qualifications required for mid-tier veterinary professionals and the types of services they might be able to perform (28) as it is plausible that knowing the potential risks associated with having different services provided by professionals other than veterinarians might influence people’s willingness to obtain care from such professionals. However, no matter how carefully worded, providing such detailed information could also have skewed participants’ responses. We therefore assumed the risk of a more neutral presentation, but we acknowledge the possibility that not detailing the complexities associated with mid-tier professionals could have favorably biased perceptions of them. Our results should therefore be interpreted with these considerations in mind.

The specific targeting of individuals within the households responsible for seeking veterinary care was a key strength that also introduced some limitations. By focusing on those who reported having the primary responsibility of seeking care, we ensured that our data more accurately reflected the experiences and barriers faced by U.S. pet owners in accessing veterinary care, which enhances the likelihood of real-world validity of our findings. However, the resulting subsamples were substantially smaller than the bigger population initially targeted, hindering our abilities to explore certain relationships between variables such as primary care-seekers’ demographics and their perceived access to care. As noted previously, one approach that could avoid this limitation is broadening recruitment strategies or extending the survey administration period. Another option would be to recruit care-seekers to participate in focus groups or in-depth interviews to facilitate further exploration of topics that our survey design could not capture.

Future research is needed to identify which demographics are most impacted by various barriers to veterinary care and which strategies are most effective in overcoming these challenges to protect animal health and welfare. It is also crucial to gain further understanding of the factors contributing to the observed distrust and dissatisfaction with veterinarians. Investigating these topics is essential to developing effective solutions that minimize care gaps for pet families, enhance quality of care, and ensure positive animal welfare outcomes.

## 5 Conclusion

This study revealed new insights related to U.S. pet owners’ perceptions of access to veterinary care. Focusing on the specific subsamples of caregivers and care-seekers enabled us to capture the direct experiences of those actively seeking veterinary services that may be obscured in surveys that broadly target the population of pet owners, regardless of their role. While most reported having ‘access’, a considerable proportion still encountered barriers and care-seeking experiences that were unsatisfactory. Demographic factors, including age, education level, and income level were found to influence perceptions of alternative veterinary service providers and potential care-seeking behaviors. Our findings also identified trust in veterinarians as a critical area for improvement. Strengthening trust within the veterinarian-client relationship is therefore necessary to enhance the experience of the client and ensure future care-seeking decisions that support good animal welfare outcomes. These findings have practical implications for various stakeholders and policymakers, emphasizing that strategies to improve access to veterinary care should account for the social and relational factors that shape pet owners’ experiences.

Whether or not they had easy access to care, more respondents were willing to seek care from alternative providers than forego it, suggesting that there is a need to address some care-seekers’ desire for a broad range of care options. Failure to meet this need could have implications for animal welfare, as pet owners may turn to unqualified or unreliable sources to guide their care decisions. However, it remains unknown why some care-seekers who reported limited access to care were less willing to consider using alternative care providers. Future research is needed to determine if these pet owners prefer different solutions or are simply forgoing care altogether. It is also unclear whether specific barriers influence care-seeking behavior more than others, and if these barriers may differ between demographic groups. Understanding these nuances and regional variations in care constraints, could provide critical guidance to the veterinary community on how to address the most pressing obstacles to equitable, high-quality veterinary care. Ultimately, this study highlights the need to build on its findings and incorporate them into considerations of interventions that may enhance both access to care and willingness to seek it.

## Data Availability

The original contributions presented in the study are included in the article/Supplementary material, further inquiries can be directed to the corresponding author.
