# Supplementary material for: U.S. residents’ self-reported access to veterinary care and implications for care-seeking decisions
Source: Front Vet Sci. 2025 Nov 6;12:1655537. doi: 10.3389/fvets.2025.1655537 (PMC12631267; doi:10.3389/fvets.2025.1655537)
Supplement: Supplementary file 1 [file Data_Sheet_1.docx]

**Questions Analyzed**

*Participants were presented with the research participant consent form approved by IRB*

Start of Block: Demographics Part A

Q1 I am:

- Female (1)
- Male (2)

Q2 I am ___ years old.

- Under 18 (1)
- 18-24 (2)
- 25-34 (3)
- 35-44 (4)
- 45-54 (5)
- 55-64 (6)
- 65 + (7)

Q3 My region of residence is: ________. (Please select one option from the drop down menu.)

▼ Northeast (CT, ME, MA, NH, NJ, NY, PA, RI, VT) (1) ... West (AK, AZ, CA, CO, HI, ID, MT, NV, NM, OR, UT, WA, WY) (4)

Q5 The best description of my educational background is (Please select from the following):

- Did not graduate from high school (1)
- Graduated from high school, did not attend college (2)
- Attended college, no degree earned (3)
- Attended college, bachelor's (B.A. or B.S.), associate's, or trade degree earned (4)
- Graduate or advanced degree earned (M.S., Ph.D., Law school) (5)

Q6 My annual pre-tax, household income is (Please select from the following):

- 0 - $24,999 (1)
- $25,000 - $49,999 (2)
- $50,000 - $74,999 (3)
- $75,000 - $99,999 (4)
- $100,000 + (5)

Start of Block: Ownership History

Q7 How many pets do you currently own?

- 0 (1)
- 1 (2)
- 2 (3)
- 3 or more (4)
- I do not currently own a pet but I plan to obtain one within the next 12 months (5)

Q8 What pet(s) do you currently own? (Please select all that apply):

- Dog(s) (1)
- Cat(s) (2)
- Rabbit(s)/Guinea Pig(s)/Chinchilla(s) (3)
- Small Rodent(s) (4)
- Reptile(s)/Amphibian(s) (5)
- Bird(s) (6)
- Fish (7)
- Other (8) __________________________________________________
- Not applicable (10)

Display This Question:

If How many pets do you currently own? = I do not currently own a pet but I plan to obtain one within the next 12 months

Q9 What pet(s) do you plan to own within the next 12 months? (Please select all that apply):

- Dog(s) (1)
- Cat(s) (2)
- Rabbit(s)/Guinea Pig(s)/Chinchilla(s) (3)
- Small Rodent(s) (4)
- Reptile(s)/Amphibian(s) (5)
- Bird(s) (6)
- Fish (7)
- Other (8) __________________________________________________
- Not applicable (9)

Display This Question:

If How many pets do you currently own? = 0

Q10 If you do not currently own pets, have you owned pet(s) within the last 5 years?

- Yes (1)
- No (2)

Display This Question:

If If you do not currently own pets, have you owned pet(s) within the last 5 years? = Yes

Q11 What pet(s) have you owned within the past 5 years? (Please select all that apply):

- Dog(s) (1)
- Cat(s) (2)
- Rabbit(s)/Guinea Pig(s)/Chinchilla(s) (3)
- Small Rodent(s) (4)
- Reptile(s)/Amphibian(s) (5)
- Bird(s) (6)
- Fish (7)
- Other (8) __________________________________________________

Display This Question:

If How many pets do you currently own? ≠ 0

Or If you do not currently own pets, have you owned pet(s) within the last 5 years? = Yes

Q12 I am the ____ . (Please select all that apply):

- Primary caregiver for my pet(s) (3)
- Primary care seeker for veterinary care (4)
- Neither (5)

Start of Block: Perceptions of Access to Veterinary Care and Veterinary Care Experiences

Q16 I believe that the term 'access to veterinary care' implies... (Please rate your level of agreement or disagreement with the options below on a scale of [1] strongly disagree to [5] strongly agree. You may provide a response in the 'other' text box, however, you are not obligated to do so.)

|  | [1] Strongly Disagree (1) | [2] Disagree (2) | [3] Neutral (3) | [4] Agree (4) | [5] Strongly Agree (5) |
| --- | --- | --- | --- | --- | --- |
| Affordability (1) |  |  |  |  |  |
| Close geographic proximity (2) |  |  |  |  |  |
| Availability of service providers (3) |  |  |  |  |  |
| Ease of communication (4) |  |  |  |  |  |
| Disability accommodations for client (5) |  |  |  |  |  |
| Other (6) |  |  |  |  |  |

Display This Question:

If I am the ____ . (Please select all that apply): = Primary care seeker for veterinary care

Q20 I seek out my veterinarian for the following types of care (Please select all that apply. You may provide a response in the 'other' text box, however, you are not obligated to do so.):

- Preventative care (e.g., annual examinations, dental care, vaccinations, parasite control) (1)
- Treatment (e.g., illness, injury, emergency care) (2)
- Reproductive care (e.g., genetic health testing, breeding, whelping/giving birth) (3)
- Behavioral consultations (4)
- Other (5) __________________________________________________

Display This Question:

If I am the ____ . (Please select all that apply): = Primary care seeker for veterinary care

Q18 My veterinarian appropriately meets my need for accommodations (e.g., ramps, enlarged print, interpreter, etc.).

- Yes (1)
- No (2)
- Not applicable (3)

Display This Question:

If I am the ____ . (Please select all that apply): = Primary care seeker for veterinary care

| 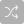 |
| --- |

Q83 Please rate your level of agreement or disagreement with the statements below on a scale of [1] strongly disagree to [5] strongly agree.

|  | [1] Strongly Disagree (1) | [2] Disagree (2) | [3] Neutral (3) | [4] Agree (4) | [5] Strongly Agree (5) |
| --- | --- | --- | --- | --- | --- |
| In the area in which I live, it is easy for me to access veterinary care. (1) |  |  |  |  |  |
| I typically see my veterinarian at least once a year (2) |  |  |  |  |  |
| I would prefer to have more frequent visits with my veterinarian (3) |  |  |  |  |  |
| I would prefer to have less frequent visits with my veterinarian (4) |  |  |  |  |  |
| It takes an unnecessarily long time to get an appointment with my veterinarian. (9) |  |  |  |  |  |
| My veterinary visits are too long (5) |  |  |  |  |  |
| My veterinary visits are too short (6) |  |  |  |  |  |
| During vet appointments, I wish I had more time with my veterinarian. (8) |  |  |  |  |  |
| My veterinarian spends an adequate amount of time with me (10) |  |  |  |  |  |

Display This Question:

If I am the ____ . (Please select all that apply): = Primary care seeker for veterinary care

| 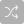 |
| --- |

Q22 Please rate your level of agreement or disagreement with the statements below on a scale of [1] strongly disagree to [5] strongly agree.

|  | [1] Strongly Disagree (1) | [2] Disagree (2) | [3] Neutral (3) | [4] Agree (4) | [5] Strongly Agree (5) |
| --- | --- | --- | --- | --- | --- |
| I am reluctant to seek veterinary care. (1) |  |  |  |  |  |
| I have had to forego seeking veterinary care in order to meet other pressing needs (e.g., my own medical care). (15) |  |  |  |  |  |
| Before going to the veterinarian, I would rather search online or ask a friend for a solution. (2) |  |  |  |  |  |
| I have difficulty communicating with my veterinarian. (3) |  |  |  |  |  |
| My veterinarian makes me feel comfortable when I ask questions. (4) |  |  |  |  |  |
| My veterinarian answers my questions in a way I can understand. (5) |  |  |  |  |  |
| My veterinarian interacts with me in a way that is culturally sensitive (16) |  |  |  |  |  |
| I feel respected by my veterinarian (6) |  |  |  |  |  |
| I feel that my veterinarian really listens to my concerns (17) |  |  |  |  |  |
| I believe my veterinarian possesses the necessary tools and expertise to treat my animal(s). (12) |  |  |  |  |  |
| I am dissatisfied with the veterinarian/veterinary care options accessible to me, but they are my only choice (18) |  |  |  |  |  |
| I trust my veterinarian and believe they provide the best possible care. (13) |  |  |  |  |  |

Start of Block: Perceptions of Veterinary Service Providers and Care Preferences

Display This Question:

If I am the ____ . (Please select all that apply): = Primary care seeker for veterinary care

Q92 Please rate the frequency with which you experience the following conditions on a scale of [1] never to [5] always.

|  | [1] Never (1) | [2] Rarely (2) | [3] Sometimes (3) | [4] Often (4) | [5] Always (5) |
| --- | --- | --- | --- | --- | --- |
| I am satisfied with both the quality of care my veterinarian provides my pet and the interactions they have with me (1) |  |  |  |  |  |
| I am satisfied with the quality of care my veterinarian provides my pet, but I am dissatisfied with the interactions they have with me (2) |  |  |  |  |  |
| I am dissatisfied with the quality of care my veterinarian provides my pet, but satisfied with their interactions with me (3) |  |  |  |  |  |
| I am dissatisfied with both the quality of care my veterinarian provides my pet and their interactions with me (4) |  |  |  |  |  |

Display This Question:

If The following factors create difficulty for me when I need to seek veterinary care. (Please rate... = [5] Strongly Agree

Or The following factors create difficulty for me when I need to seek veterinary care. (Please rate... = [4] Agree

Or The following factors create difficulty for me when I need to seek veterinary care. (Please rate... = [3] Neutral

| 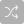 |
| --- |

Q86 Please rate your level of agreement or disagreement with the statements below on a scale of [1] strongly disagree to [5] strongly agree.

|  | [1] Strongly Disagree (1) | [2] Disagree (2) | [3] Neutral (3) | [4] Agree (4) | [5] Strongly Agree (5) |
| --- | --- | --- | --- | --- | --- |
| In circumstances where I experience barriers to accessing a veterinarian, I would be willing to see a veterinary technician/veterinary nurse (5) |  |  |  |  |  |
| In circumstances where I experience barriers to accessing a veterinarian, I would be willing to see a mid-tier veterinary professional (more advanced education/training than a veterinary technician; similar to a nurse practitioner or physician's assistant in human medicine) (6) |  |  |  |  |  |
| In circumstances where I experience barriers to accessing a veterinarian, I would prefer to see a veterinary technician/veterinary nurse rather than forego care (8) |  |  |  |  |  |
| In circumstances where I experience barriers to accessing a veterinarian, I would prefer to see a mid-tier veterinary professional (more advanced education/training than a veterinary technician; similar to a nurse practitioner or physician's assistant in human medicine) rather than forego care (9) |  |  |  |  |  |

Display This Question:

If How many pets do you currently own? ≠ 0

Or If you do not currently own pets, have you owned pet(s) within the last 5 years? = Yes

| 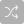 |
| --- |

Q90 Please rate your level of agreement or disagreement with the statements below on a scale of [1] strongly disagree to [5] strongly agree.

|  | [1] Strongly Disagree (1) | [2] Disagree (2) | [3] Neutral (3) | [4] Agree (4) | [5] Strongly Agree (5) |
| --- | --- | --- | --- | --- | --- |
| I believe a well-trained veterinary technician/veterinary nurse can provide just as high-quality service as a veterinarian (1) |  |  |  |  |  |
| I believe a well-trained mid-tier veterinary professional (more advanced education/training than a veterinary technician; similar to a nurse practitioner or physician's assistant in human medicine) can provide just as high-quality service as a veterinarian (2) |  |  |  |  |  |
| I would choose a competent veterinary technician/veterinary nurse whose demographics more closely matches mine than a veterinarian with whom there is a significant mismatch (4) |  |  |  |  |  |
| I would choose a competent mid-tier veterinary professional (more advanced education/training than a veterinary technician; similar to a nurse practitioner or physician's assistant in human medicine) whose demographics more closely matches mine than a veterinarian with whom there is a significant mismatch (5) |  |  |  |  |  |
| I would choose a competent veterinary technician/veterinary nurse whose cultural sensitivity is higher than the veterinarian I currently see (7) |  |  |  |  |  |
| I would choose a competent mid-tier veterinary professional (more advanced education/training than a veterinary technician; similar to a nurse practitioner or physician's assistant in human medicine) whose cultural sensitivity is higher than the veterinarian I currently see (8) |  |  |  |  |  |

Start of Block: Demographics Part B

Q30 I am _____ . (Please select from the following):

- White (1)
- Black or African American (2)
- Asian (3)
- American Indian or Alaskan Native (4)
- Pacific Islander or Native Hawaiian (5)
- Other: (6) __________________________________________________
- Prefer not to answer. (7)

Q31 My ethnicity is of Hispanic, Latino, or Spanish origin.

- Yes (1)
- No (2)
- Prefer not to answer. (3)

Q32 I am able to communicate without difficulty or need for assistance in the following language(s). (Please select from the following

- English (1)
- Spanish (2)
- Other (3) __________________________________________________
- Prefer not to answer (4)

Q33 I require accommodations to adequately access veterinary care. (Please select all that apply):

- Physical (e.g., ramps, additional handrails, elevators) (1)
- Neurological (e.g., provision of oral and written information) (2)
- Cognitive (e.g., memory aids such as checklists) (3)
- Emotional (e.g. predictable routine, positive reinforcement) (4)
- Visual (e.g., enlarged print or Braille) (5)
- Auditory (e.g., visual cues, interpreter) (6)
- Language translation (7)
- Not applicable (8)
- Prefer not to answer (9)
