# Supplementary material for: U.S. residents’ self-reported access to veterinary care and implications for care-seeking decisions
Source: Front Vet Sci. 2025 Nov 6;12:1655537. doi: 10.3389/fvets.2025.1655537 (PMC12631267; doi:10.3389/fvets.2025.1655537)
Supplement: Supplementary file 3 [file Data_Sheet_3.docx]

Supplementary Material

# Supplementary Tables

**Table S1:** Demographic characteristics of respondents for surveys that were both entirely and partially completed.

| Demographic Variable | Completed Surveys  (%) | Partially Completed Surveys  (%) | US Census  (%) |
| --- | --- | --- | --- |
| Sex | **(n = 1177)** | **(n = 1525)** |  |
| Male^*^ | 43.5^ᴪ^ | 44.9 | 49.3 |
| Female | 56.5 | 55.1 | 50.7 |
|  |  |  |  |
| Age | **(n = 1177)** | **(n = 1525)** |  |
| 18-24 | 14.4 | 12.3 | 11.4 |
| 25-34 | 14.9 | 22.8 | 17.5 |
| 35-44 | 21.6 | 19.2 | 16.9 |
| 45-54 | 20.1 | 17.6 | 15.7 |
| 55-64 | 16.6 | 17.2 | 16.5 |
| 65+ | 12.5^ᴪ^ | 10.9 ^ᴪ^ | 22.0 |
|  |  |  |  |
| Region | **(n = 1177)** | **(n = 1525)** |  |
| Northeast | 19.7 | 17.8 | 17.0 |
| South | 37.8 | 40.3 | 38.9 |
| Midwest | 20.5 | 21.2 | 20.6 |
| West | 22.0 | 20.7 | 23.6 |
|  |  |  |  |
| Education Level | **(n = 1177)** | **(n = 1383)** |  |
| Did not graduate high school | 3.3^ᴪ^ | 3.1^ᴪ^ | 9.6 |
| High school graduate, no college | 26.0 | 26.1 | 29.2 |
| Attended college, no degree earned | 21.0 | 24.1 | 16.5 |
| Attended college, bachelor’s (B.A./B.S.), associates, or trade degree earned | 36.3 | 34.3 | 32.0 |
| Graduate or advanced degree earned (M.S., Ph.D., Law School) | 13.4 | 12.4 | 12.7 |
|  |  |  |  |
| Income Level | **(n = 1177)** | **(n = 1383)** |  |
| $0 - $24,999 | 19.5 | 18.9 | 15.8 |
| $25,000 - $49,999 | 23.2 | 23.4 | 18.2 |
| $50,000 - $74,999 | 18.9 | 18.7 | 16.2 |
| $75,000 - $99,999 | 15.7 | 16.7 | 12.3 |
| $100,000+ | 22.8^ᴪ^ | 22.3^ᴪ^ | 37.5 |

^ᴪ Indicates that the proportion of respondents is statistically different than the US Census at the 0.05 level.^

^* The male demographic group in the subsample of completed surveys is not statistically proportional to the U.S. Census data. However, when partially completed surveys are included in the analysis, the male demographic becomes statistically representative of the U.S. Census at the 0.05 level.^

**Table S2.** Results of Chi-square test of independence for relationships between respondents’ demographics, perceptions of access to veterinary care, beliefs about alternative veterinary service providers, and preferences for and willingness to utilize alternative veterinary service providers.

|  |  | Sex | Age | Region | Education | Income |
| --- | --- | --- | --- | --- | --- | --- |
| In the area in which I live, it is easy for me to access veterinary care (n = 206)^a^ | Χ^2^ (df) | 0.61 (1) | 0.70 (3) | 6.39 (3) | 1.50 (3) | 1.71 (3) |
|  | p-value | 0.435 | 0.873 | **0.044*** | 0.713* | 0.665* |
| I believe a well-trained veterinary technician/veterinary nurse can provide just as high-quality service as a veterinarian.  (n = 788)^b^ | Χ^2^ (df) | 3.04 (1) | 31.81 (3) | 0.88 (3) | 3.85 (3) | 10.12 (3) |
|  | p-value | 0.081 | **< 0.001** | 0.829 | 0.278 | **0.018** |
| I believe a well-trained mid-tier veterinary professional can provide just as high-quality service as a veterinarian. (n = 764) ^b^ | Χ^2^ (df) | < 0.10 (1) | 7.79 (3) | 1.43 (3) | 3.53 (3) | 8.20 (3) |
|  | p-value | 0.957 | **0.051** | 0.699 | 0.318 | **0.042** |
| I would choose a competent veterinary technician/veterinary nurse whose demographics more closely matches mine than a veterinarian with whom there is a significant mismatch. (n = 716) ^b^ | Χ^2^ (df) | < 0.10 (1) | 10.93 (3) | 1.41 (3) | 8.65 (3) | 13.02 (3) |
|  | p-value | 0.965 | **0.012** | 0.703 | **0.034** | **0.005** |
| I would choose a competent mid-tier veterinary professional whose demographics more closely matches mine than a veterinarian with whom there is a significant mismatch. (n = 712) ^b^ | Χ^2^ (df) | < 0.10 (1) | 9.03 (3) | 1.45 (3) | 10.01 (3) | 17.22 (3) |
|  | p-value | 0.946 | **0.029** | 0.694 | **0.019** | **< 0.001** |
| I would choose a competent veterinary technician/veterinary nurse whose cultural sensitivity is higher than the veterinarian I currently see. (n = 715) ^b^ | Χ^2^ (df) | 0.12 (1) | 36.16 (3) | 5.45 (3) | 6.48 (3) | 19.60 (3) |
|  | p-value | 0.734 | **< 0.001** | 0.141 | 0.090 | **< 0.001** |
| I would choose a competent mid-tier veterinary professional whose cultural sensitivity is higher than the veterinarian I currently see. (n = 703) ^b^ | Χ^2^ (df) | 0.15 (1) | 23.76 (3) | 2.75 (3) | 5.20 (3) | 19.70 (3) |
|  | p-value | 0.704 | **< 0.001** | 0.433 | 0.158 | **< 0.001** |
| In circumstances where I experience barriers to accessing a veterinarian, I would be willing to see a veterinary technician/veterinary nurse. (n = 166)^c^ | Χ^2^ (df) | 0.18 (1) | 4.41 (3) | 6.03 (3) | 0.248 (3) | 1.64 (3) |
|  | p-value | 0.668 | 0.221 | 0.110 | 0.969 | 0.651 |
| In circumstances where I experience barriers to accessing a veterinarian, I would be willing to see a mid-tier veterinary professional. (n = 151) ^c^ | Χ^2^ (df) | 2.84 (1) | 5.68 (3) | 2.37 (3) | 1.82 (3) | 1.08 (3) |
|  | p-value | 0.092 | 0.089* | 0.516* | 0.664* | 0.781* |
| In circumstances where I experience barriers to accessing a veterinarian, I would prefer to see a veterinary technician/veterinary nurse rather than forego care. (n = 159) ^c^ | Χ^2^ (df) | 0.12 (1) | 11.41 (3) | 2.20 (3) | 0.93 (3) | 0.82 (3) |
|  | p-value | 0.722 | **0.010** | 0.535* | 0.834* | 0.859* |
| In circumstances where I experience barriers to accessing a veterinarian, I would prefer to see a mid-tier veterinary professional rather than forego care. (n = 156) ^c^ | Χ^2^ (df) | 0.12 (1) | 1.29 (3) | 2.14 (3) | 2.41 (3) | 4.69 (3) |
|  | p-value | 0.738 | 0.733 | 0.544 | 0.493 | 0.196 |

^a Indicates that these questions were presented to the subsample of 236 participants who identified as the primary care-seeker in their household for veterinary care.^

^b Indicates that these questions were presented to the subsample of 1026 participants who reported current, recent, or future pet ownership.^

^c Indicated that these questions were presented to the subsample of 210 primary care-seekers for veterinary care and reported experiencing at least one barrier to accessing veterinary care.^

^The number of participants in each subsample who responded to each statement is shown in the table.^

^*Indicates that the p-value was calculated using the Fisher’s exact test.^

^Statistically significant associations at the 0.05 level are shown in bolded text.^

**Table S3.** Results of Chi-square test of independence for relationships between respondents’ perceptions of access to veterinary care, beliefs about alternative veterinary service providers, and preferences for and willingness to utilize alternative veterinary service providers

|  |  | In the area in which I live, it is easy for me to access veterinary care | |
| --- | --- | --- | --- |
| I believe a well-trained veterinary technician/veterinary nurse can provide just as high-quality service as a veterinarian.  (n = 166)^a^ | Χ^2^ (df) | 3.52 (1) |  |
|  | p-value | 0.073* |  |
| I believe a well-trained mid-tier veterinary professional can provide just as high-quality service as a veterinarian. (n = 150) ^a^ | Χ^2^ (df) | 1.82 (1) |  |
|  | p-value | 0.242* |  |
| I would choose a competent veterinary technician/veterinary nurse whose demographics more closely matches mine than a veterinarian with whom there is a significant mismatch. (n = 134) ^a^ | Χ^2^ (df) | 1.47 (1) |  |
|  | p-value | 0.227* |  |
| I would choose a competent mid-tier veterinary professional whose demographics more closely matches mine than a veterinarian with whom there is a significant mismatch. (n = 130) ^a^ | Χ^2^ (df) | 3.17 (1) |  |
|  | p-value | 0.088* |  |
| I would choose a competent veterinary technician/veterinary nurse whose cultural sensitivity is higher than the veterinarian I currently see. (n = 133) ^a^ | Χ^2^ (df) | 2.35 (1) |  |
|  | p-value | 0.125 |  |
| I would choose a competent mid-tier veterinary professional whose cultural sensitivity is higher than the veterinarian I currently see. (n = 119) ^a^ | Χ^2^ (df) | 4.28 (1) |  |
|  | p-value | 0.052* |  |
| In circumstances where I experience barriers to accessing a veterinarian, I would be willing to see a veterinary technician/veterinary nurse. (n = 150) ^b^ | Χ^2^ (df) | 5.08 (1) |  |
|  | p-value | **0.036*** |  |
| In circumstances where I experience barriers to accessing a veterinarian, I would be willing to see a mid-tier veterinary professional. (n = 138) ^b^ | Χ^2^ (df) | 3.98 (1) |  |
|  | p-value | 0.069* |  |
| In circumstances where I experience barriers to accessing a veterinarian, I would prefer to see a veterinary technician/veterinary nurse rather than forego care. (n = 144) ^b^ | Χ^2^ (df) | 5.79 (1) |  |
|  | p-value | **0.031*** |  |
| In circumstances where I experience barriers to accessing a veterinarian, I would prefer to see a mid-tier veterinary professional rather than forego care. (n = 140) ^b^ | Χ^2^ (df) | 7.24 (1) |  |
|  | p-value | **0.016*** |  |

^a Indicates that these questions were presented to the subsample of 1026 participants who reported current, recent, or future pet ownership and who also reported being a primary care-seeker for veterinary care.^

^b Indicated that these questions were presented to the subsample of 210 primary care-seekers for veterinary care and reported experiencing at least one barrier to accessing veterinary care.^

^The number of participants in each subsample who responded to each statement is shown in the table.^

^*Indicates that the p-value was calculated using the Fisher’s exact test.^

^Statistically significant associations at the 0.05 level are shown in bolded text.^

**Table S4**. Cross-tabulations between participants’ demographics (sex, age, region) and their perceptions of access to care and alternative veterinary service providers without collapsed categories.

|  |  | Sex | | Age | | | | | Region | | | | | |
| --- | --- | --- | --- | --- | --- | --- | --- | --- | --- | --- | --- | --- | --- | --- |
|  |  | Male  (n, %) | Female  (n, %) | 18-24  (n, %) | 25-34  (n, %) | 35-44  (n, %) | 45-54  (n, %) | 55-64  (n, %) | | 65+  (n, %) | Northeast (n, %) | South  (n, %) | Midwest (n, %) | West  (n, %) |
| In the area in which I live, it is easy for me to access veterinary care (n = 234)^a^ | Strongly Agree | 36 (35.6) | 51 (38.3) | 7 (26.9) | 11 (33.3) | 23 (42.6) | 15 (34.9) | 15 (38.5) | | 16 (41.0) | 19 (45.2) | 30 (36.1) | 16 (38.1) | 22 (32.8) |
|  | Agree | 47 (46.5) | 50 (37.6) | 14 (53.8) | 14 (42.4) | 21 (38.9) | 14 (32.6) | 18 (46.2) | | 16 (41.0) | 20 (47.6) | 35 (42.2) | 13 (31.0) | 29 (43.3) |
|  | Neutral | 10 (9.9) | 18 (13.5) | 4 (15.4) | 4 (12.1) | 5 (9.3) | 9 (20.9) | 3 (7.7) | | 3 (7.7) | 3 (7.1) | 7 (8.4) | 8 (19.0) | 10 (14.9) |
|  | Disagree | 3 (3.0) | 10 (7.5) | 0 (0.0) | 3 (9.1) | 2 (3.7) | 4 (9.3) | 2 (5.1) | | 2 (5.1) | 0 (0.0) | 6 (7.2) | 2 (4.8) | 5 (7.5) |
|  | Strongly Disagree | 5 (5.0) | 4 (3.0) | 1 (3.8) | 1 (3.0) | 3 (5.6) | 1 (2.3) | 1 (2.6) | | 2 (5.1) | 0 (0.0) | 5 (6.0) | 3 (7.1) | 1 (1.5) |
| I believe a well-trained veterinary technician/veterinary nurse can provide just as high-quality service as a veterinarian  (n = 1025) ^b^ | Strongly Agree | 98 (22.7) | 157 (26.5) | 46 (29.1) | 52 (31.9) | 77 (32.6) | 44 (21.7) | 26 (16.0) | | 10 (9.8) | 56 (28.6) | 91 (23.5) | 47 (22.7) | 61 (26.0) |
|  | Agree | 185 (42.8) | 243 (41.0) | 62 (39.2) | 77 (47.2) | 95 (40.3) | 84 (41.4) | 65 (39.9) | | 45 (44.1) | 77 (39.3) | 163 (42.1) | 87 (42.0) | 101 (43.0) |
|  | Neutral | 96 (22.2) | 141 (23.8) | 39 (24.7) | 25 (15.3) | 47 (19.9) | 55 (27.1) | 44 (27.0) | | 27 (26.5) | 44 (22.4) | 93 (24.0) | 49 (23.7) | 51 (21.7) |
|  | Disagree | 42 (9.7) | 38 (6.4) | 8 (5.1) | 8 (4.9) | 12 (5.1) | 16 (7.9) | 22 (13.5) | | 14 (13.7) | 13 (6.6) | 32 (8.3) | 17 (8.2) | 18 (7.7) |
|  | Strongly Disagree | 11 (2.5) | 14 (2.4) | 3 (1.9) | 1 (0.6) | 5 (2.1) | 4 (2.0) | 6 (3.7) | | 6 (5.9) | 6 (3.1) | 8 (2.1) | 7 (3.4) | 4 (1.7) |
| I believe a well-trained mid-tier veterinary professional can provide just as high-quality service as a veterinarian.  (n = 1024) ^b^ | Strongly Agree | 88 (20.4) | 152 (25.6) | 40 (25.3) | 46 (28.4) | 81 (34.3) | 41 (20.2) | 26 (16.0) | | 6 (5.9) | 48 (24.6) | 93 (24.0) | 39 (18.8) | 60 (25.5) |
|  | Agree | 192 (44.5) | 235 (39.6) | 61 (38.6) | 75 (46.3) | 88 (37.3) | 88 (43.3) | 65 (39.9) | | 50 (49.0) | 78 (40.0) | 163 (42.1) | 92 (44.4) | 94 (40.0) |
|  | Neutral | 110 (25.5) | 150 (25.3) | 42 (26.6) | 28 (17.3) | 50 (21.2) | 55 (27.1) | 50 (30.7) | | 35 (34.3) | 52 (26.7) | 91 (23.5) | 54 (26.1) | 63 (26.8) |
|  | Disagree | 32 (7.4) | 40 (6.7) | 8 (5.1) | 12 (7.4) | 13 (5.5) | 14 (6.9) | 16 (9.8) | | 9 (8.8) | 12 (6.2) | 30 (7.8) | 15 (7.2) | 15 (6.4) |
|  | Strongly Disagree | 9 (2.1) | 16 (2.7) | 7 (4.4) | 1 (0.6) | 4 (1.7) | 5 (2.5) | 6 (3.7) | | 2 (2.0) | 5 (2.6) | 10 (2.6) | 7 (3.4) | 3 (1.3) |
| I would choose a competent veterinary technician/veterinary nurse whose demographics more closely matches mine than a veterinarian with whom there is a significant mismatch.  (n = 1024) ^b^ | Strongly Agree | 91 (21.1) | 128 (21.6) | 41 (25.9) | 38 (23.3) | 75 (31.9) | 32 (15.8) | 25 (15.3) | | 8 (7.8) | 40 (20.4) | 90 (23.3) | 40 (19.3) | 49 (20.9) |
|  | Agree | 167 (38.7) | 190 (32.0) | 55 (34.8) | 69 (42.3) | 68 (28.9) | 73 (36.0) | 53 (32.5) | | 39 (38.2) | 73 (37.2) | 126 (32.6) | 70 (33.8) | 88 (37.4) |
|  | Neutral | 110 (25.5) | 198 (33.4) | 42 (26.6) | 36 (22.1) | 69 (29.4) | 64 (31.5) | 60 (36.8) | | 37 (36.3) | 54 (27.6) | 116 (30.1) | 67 (32.4) | 71 (30.2) |
|  | Disagree | 38 (8.8) | 51 (8.6) | 14 (8.9) | 14 (8.6) | 12 (5.1) | 21 (10.3) | 15 (19.2) | | 13 (12.7) | 16 (8.2) | 36 (9.3) | 20 (9.7) | 17 (7.2) |
|  | Strongly Disagree | 25 (5.8) | 26 (5.4) | 6 (3.8) | 6 (3.7) | 11 (4.7) | 13 (6.4) | 10 (6.1) | | 5 (4.9) | 13 (6.6) | 18 (4.7) | 10 (4.8) | 10 (4.3) |
| I would choose a competent mid-tier veterinary professional whose demographics more closely matches mine than a veterinarian with whom there is a significant mismatch.  (n = 1026) ^b^ | Strongly Agree | 99 (22.9) | 112 (18.9) | 38 (24.1) | 37 (22.7) | 67 (28.3) | 39 (19.2) | 23 (14.1) | | 7 (6.9) | 40 (20.4) | 80 (20.7) | 38 (18.4) | 53 (22.5) |
|  | Agree | 156 (36.1) | 216 (36.4) | 55 (34.8) | 72 (44.2) | 81 (34.2) | 65 (32.0) | 56 (34.4) | | 43 (42.2) | 67 (34.2) | 138 (35.7) | 78 (37.7) | 89 (37.7) |
|  | Neutral | 121 (28.0) | 193 (32.5) | 42 (26.6) | 47 (28.8) | 59 (24.9) | 67 (33.0) | 61 (37.4) | | 38 (37.3) | 68 (34.7) | 120 (31.0) | 60 (29.0) | 66 (28.0) |
|  | Disagree | 35 (8.1) | 51 (8.6) | 14 (8.9) | 5 (3.1) | 19 (8.0) | 22 (10.8) | 16 (9.8) | | 10 (9.8) | 13 (6.6) | 32 (8.3) | 21 (10.1) | 20 (8.5) |
|  | Strongly Disagree | 21 (4.9) | 22 (3.7) | 9 (5.7) | 2 (1.2) | 11 (4.6) | 10 (4.9) | 7 (4.3) | | 4 (3.9) | 8 (4.1) | 17 (4.4) | 10 (4.8) | 8 (3.4) |
| I would choose a competent veterinary technician/veterinary nurse whose cultural sensitivity is higher than the veterinarian I currently see.  (n = 1024) ^b^ | Strongly Agree | 97 (22.5) | 132 (22.3) | 33 (20.9) | 41 (25.3) | 82 (34.7) | 42 (20.7) | 24 (14.7) | | 7 (6.9) | 41 (21.0) | 92 (23.8) | 40 (19.3) | 56 (23.8) |
|  | Agree | 148 (34.3) | 195 (32.9) | 59 (37.3) | 68 (42.0) | 73 (30.9) | 68 (33.5) | 49 (30.1) | | 26 (25.5) | 64 (32.8) | 127 (32.8) | 67 (32.4) | 85 (36.2) |
|  | Neutral | 127 (29.5) | 182 (30.7) | 45 (28.5) | 38 (23.5) | 64 (27.1) | 58 (28.6) | 55 (33.7) | | 49 (48.0) | 60 (30.8) | 116 (30.0) | 64 (30.9) | 69 (29.4) |
|  | Disagree | 37 (8.6) | 54 (9.1) | 13 (8.2) | 11 (6.8) | 12 (5.1) | 25 (12.3) | 22 (13.5) | | 8 (7.8) | 17 (8.7) | 36 (9.3) | 24 (11.6) | 14 (6.0) |
|  | Strongly Disagree | 22 (5.1) | 30 (5.1) | 8 (5.1) | 4 (2.5) | 5 (2.1) | 10 (4.9) | 13 (8.0) | | 12 (11.8) | 13 (6.7) | 16 (4.1) | 12 (5.8) | 11 (4.7) |
| I would choose a competent mid-tier veterinary professional whose cultural sensitivity is higher than the veterinarian I currently see.  (n = 1025) ^b^ | Strongly Agree | 101 (23.4) | 125 (21.1) | 33 (20.9) | 44 (27.0) | 81 (34.3) | 35 (17.2) | 25 (15.3) | | 8 (7.8) | 44 (22.4) | 98 (25.3) | 38 (18.4) | 46 (19.6) |
|  | Agree | 160 (37.0) | 197 (33.2) | 63 (39.9) | 66 (40.5) | 74 (31.4) | 71 (35.0) | 49 (30.1) | | 34 (33.3) | 76 (38.8) | 119 (30.7) | 69 (33.3) | 93 (39.6) |
|  | Neutral | 115 (26.6) | 207 (34.9) | 43 (27.2) | 43 (26.4) | 61 (25.8) | 73 (36.0) | 58 (35.6) | | 44 (43.1) | 50 (25.5) | 124 (32.0) | 73 (35.3) | 75 (31.9) |
|  | Disagree | 32 (7.4) | 41 (6.9) | 13 (8.2) | 7 (4.3) | 12 (5.1) | 15 (7.4) | 20 (12.3) | | 6 (5.9) | 16 (8.2) | 28 (7.2) | 18 (8.7) | 11 (4.7) |
|  | Strongly Disagree | 24 (5.6) | 23 (3.9) | 6 (3.8) | 3 (1.8) | 8 (3.4) | 9 (4.4) | 11 (6.7) | | 10 (9.8) | 10 (5.1) | 18 (4.7) | 9 (4.3) | 10 (4.3) |
| In circumstances where I experience barriers to accessing a veterinarian, I would be willing to see a veterinary technician/veterinary nurse (n = 210) ^c^ | Strongly Agree | 23 (25.8) | 28 (23.1) | 10 (38.5) | 9 (28.1) | 17 (34.0) | 8 (20.5) | 4 (12.9) | | 3 (9.4) | 7 (18.9) | 23 (31.9) | 8 (21.1) | 13 (20.6) |
|  | Agree | 35 (39.3) | 52 (43.0) | 8 (30.8) | 11 (34.4) | 18 (36.0) | 14 (35.9) | 18 (28.1) | | 18 (56.3) | 23 (62.2) | 30 (41.7) | 13 (34.2) | 21 (33.3) |
|  | Neutral | 18 (20.2) | 26 (21.5) | 7 (26.9) | 8 (25.0) | 8 (16.0) | 8 (20.5) | 6 (19.4) | | 7 (21.9) | 4 (10.8) | 12 (16.7) | 9 (23.7) | 19 (30.2) |
|  | Disagree | 10 (11.2) | 9 (7.4) | 1 (3.8) | 4 (12.5) | 6 (12.0) | 5 (12.8) | 2 (6.5) | | 1 (3.1) | 2 (5.4) | 5 (6.9) | 5 (13.2) | 7 (11.1) |
|  | Strongly Disagree | 3 (3.4) | 6 (5.0) | 0 (0.0) | 0 (0.0) | 1 (2.0) | 4 (10.3) | 1 (3.2) | | 3 (9.4) | 1 (2.7) | 2 (2.8) | 3 (7.9) | 3 (4.8) |
| In circumstances where I experience barriers to accessing a veterinarian, I would be willing to see a mid-tier veterinary professional  (n = 210) ^c^ | Strongly Agree | 19 (21.1) | 28 (23.3) | 9 (34.6) | 10 (31.3) | 13 (26.0) | 8 (20.5) | 4 (12.9) | | 3 (9.4) | 10 (27.0) | 15 (20.5) | 11 (28.9) | 11 (17.7) |
|  | Agree | 30 (33.3) | 53 (44.2) | 10 (38.5) | 15 (46.9) | 15 (30.0) | 13 (33.3) | 13 (41.9) | | 17 (53.1) | 18 (48.6) | 30 (41.1) | 10 (26.3) | 25 (40.3) |
|  | Neutral | 29 (32.2) | 30 (25.0) | 5 (19.2) | 7 (21.9) | 17 (34.0) | 12 (30.8) | 11 (35.5) | | 7 (21.9) | 7 (18.9) | 19 (26.0) | 12 (31.6) | 21 (33.9) |
|  | Disagree | 7 (7.8) | 5 (4.2) | 1 (3.8) | 0 (0.0) | 3 (6.0) | 3 (7.7) | 2 (6.5) | | 3 (9.4) | 2 (5.4) | 4 (5.5) | 2 (5.3) | 4 (6.5) |
|  | Strongly Disagree | 5 (5.6) | 4 (3.3) | 1 (3.8) | 0 (0.0) | 2 (4.0) | 3 (7.7) | 1 (3.2) | | 2 (6.3) | 0 (0.0) | 5 (6.8) | 3 (7.9) | 1 (1.6) |
| In circumstances where I experience barriers to accessing a veterinarian, I would prefer to see a veterinary technician/veterinary nurse rather than forego care.  (n = 209) ^c^ | Strongly Agree | 19 (21.3) | 33 (27.5) | 12 (46.2) | 5 (15.6) | 19 (38.8) | 7 (17.9) | 7 (22.6) | | 2 (6.3) | 10 (27.0) | 21 (29.2) | 8 (21.1) | 13 (21.0) |
|  | Agree | 41 (46.1) | 45 (37.5) | 6 (23.1) | 19 (59.4) | 16 (32.7) | 15 (38.5) | 14 (45.2) | | 16 (50.0) | 18 (48.6) | 27 (37.5) | 12 (31.6) | 29 (46.8) |
|  | Neutral | 19 (21.3) | 31 (25.8) | 8 (30.8) | 7 (21.9) | 11 (22.4) | 9 (23.1) | 6 (19.4) | | 9 (28.1) | 7 (18.9) | 16 (22.2) | 13 (34.2) | 14 (22.6) |
|  | Disagree | 7 (7.9) | 7 (5.8) | 0 (0.0) | 1 (3.1) | 2 (4.1) | 6 (15.4) | 3 (9.7) | | 2 (6.3) | 2 (5.4) | 5 (6.9) | 3 (7.9) | 4 (6.5) |
|  | Strongly Disagree | 3 (3.4) | 4 (3.3) | 0 (0.0) | 0 (0.0) | 1 (2.0) | 2 (5.1) | 1 (3.2) | | 3 (9.4) | 0 (0.0) | 3 (4.2) | 2 (5.3) | 2 (3.2) |
| In circumstances where I experience barriers to accessing a veterinarian, I would prefer to see a mid-tier veterinary professional rather than forego care.  (n = 209) ^c^ | Strongly Agree | 20 (22.5) | 28 (23.3) | 4 (15.4) | 9 (28.1) | 16 (32.7) | 9 (23.1) | 6 (19.4) | | 4 (12.5) | 10 (27.0) | 18 (25.0) | 6 (15.8) | 14 (22.6) |
|  | Agree | 35 (39.3) | 45 (37.5) | 10 (38.5) | 12 (37.5) | 17 (34.7) | 14 (35.9) | 15 (48.4) | | 12 (37.5) | 17 (45.9) | 29 (40.3) | 12 (31.6) | 22 (35.5) |
|  | Neutral | 21 (23.6) | 32 (26.7) | 8 (30.8) | 9 (28.1) | 10 (20.4) | 11 (28.2) | 7 (22.6) | | 8 (25.0) | 7 (18.9) | 14 (19.4) | 14 (36.8) | 18 (29.0) |
|  | Disagree | 11 (12.4) | 12 (10.0) | 4 (15.4) | 1 (3.1) | 5 (10.2) | 4 (10.3) | 3 (9.7) | | 6 (18.8) | 3 (8.1) | 9 (12.5) | 4 (10.5) | 7 (11.3) |
|  | Strongly Disagree | 2 (2.2) | 3 (2.5) | 0 (0.0) | 1 (3.1) | 1 (2.0) | 1 (2.6) | 0 (0.0) | | 2 (6.3) | 0 (0.0) | 2 (2.8) | 2 (5.3) | 1 (1.6) |

^a Indicates that these questions were presented to the subsample of 236 participants who identified as the primary care-seeker in their household for veterinary care.^

^b Indicates that these questions were presented to the subsample of 1026 participants who reported current, recent, or future pet ownership.^

^c Indicated that these questions were presented to the subsample of 210 primary care-seekers for veterinary care and reported experiencing at least one barrier to accessing veterinary care.^

^The number of participants in each subsample who responded to each statement is shown in the table.^

**Table S5.** Cross-tabulations between participants’ demographics (education level, income level) and their perceptions of access to care and alternative veterinary service providers without collapsed categories.

|  |  | Income Level | | | | | Education Level | | | | | |
| --- | --- | --- | --- | --- | --- | --- | --- | --- | --- | --- | --- | --- |
|  |  | $0 - $24,999 (n, %) | $25, 000 - $49,999  (n, %) | $50,000 - $74,999 (n, %) | $75,000 - $99,999 (n, %) | $100,000+ (n, %) | Did not graduate high school  (n, %) | High school graduate, no college  (n, %) | Attended college, no degree earned  (n, %) | Attended college, bachelor’s (B.A./B.S.), associate’s, or trade degree earned  (n, %) | | Graduate or advanced degree earned (M.S., Ph.D., Law School)  (n, %) |
| In the area in which I live, it is easy for me to access veterinary care (n = 234)^a^ | Strongly Agree | 6 (31.6) | 12 (24.5) | 23 (50.0) | 13 (28.9) | 33 (44.0) | 6 (60.0) | 10 (25.6) | 15 (31.9) | | 38 (38.8) | 18 (45.0) |
|  | Agree | 5 (26.3) | 22 (44.9) | 16 (34.8) | 22 (48.9) | 32 (42.7) | 1 (10.0) | 19 (48.7) | 21 (44.7) | | 39 (39.8) | 17 (42.5) |
|  | Neutral | 5 (26.3) | 10 (20.4) | 4 (8.7) | 6 (13.3) | 3 (4.0) | 2 (20.0) | 7 (17.9) | 6 (12.8) | | 10 (10.2) | 3 (7.5) |
|  | Disagree | 3 (15.8) | 4 (8.2) | 1 (2.2) | 1 (2.2) | 4 (5.3) | 1 (10.0) | 3 (7.7) | 3 (6.4) | | 6 (6.1) | 0 (0.0) |
|  | Strongly Disagree | 0 (0.0) | 1 (2.0) | 2 (4.3) | 3 (6.7) | 3 (4.0) | 0 (0.0) | 0 (0.0) | 2 (4.3) | | 5 (5.1) | 2 (5.0) |
| I believe a well-trained veterinary technician/veterinary nurse can provide just as high-quality service as a veterinarian  (n = 1025)^b^ | Strongly Agree | 47 (26.3) | 59 (24.6) | 48 (24.0) | 56 (32.0) | 45 (19.5) | 13 (38.2) | 59 (21.8) | 53 (24.3) | | 110 (29.3) | 20 (15.7) |
|  | Agree | 65 (36.3) | 98 (40.8) | 96 (48.0) | 72 (41.1) | 97 (42.0) | 8 (23.5) | 114 (42.1) | 101 (46.3) | | 149 (39.7) | 56 (44.1) |
|  | Neutral | 52 (29.1) | 60 (25.0) | 39 (19.5) | 33 (18.9) | 53 (22.9) | 11 (32.4) | 73 (26.9) | 45 (20.6) | | 75 (20.0) | 33 (26.0) |
|  | Disagree | 8 (4.5) | 21 (8.8) | 11 (5.5) | 12 (6.9) | 28 (12.1) | 1 (2.9) | 17 (6.3) | 13 (6.0) | | 36 (9.6) | 13 (10.2) |
|  | Strongly Disagree | 7 (3.9) | 2 (0.8) | 6 (3.0) | 2 (1.1) | 8 (3.5) | 1 (2.9) | 8 (3.0) | 5 (3.9) | | 5 (1.3) | 5 (3.9) |
| I believe a well-trained mid-tier veterinary professional can provide just as high-quality service as a veterinarian.  (n = 1024) ^b^ | Strongly Agree | 49 (27.5) | 59 (24.6) | 36 (18.0) | 56 (32.0) | 40 (17.3) | 10 (29.4) | 56 (20.7) | 51 (23.4) | | 98 (26.1) | 25 (19.7) |
|  | Agree | 56 (31.5) | 94 (39.2) | 100 (50.0) | 77 (44.0) | 100 (43.3) | 9 (26.5) | 108 (40.0) | 95 (43.6) | | 159 (42.4) | 56 (44.1) |
|  | Neutral | 57 (32.0) | 64 (26.7) | 49 (24.5) | 30 (17.1) | 60 (26.0) | 11 (32.4) | 83 (30.7) | 47 (21.6) | | 89 (23.7) | 30 (23.6) |
|  | Disagree | 10 (5.6) | 17 (7.1) | 11 (5.5) | 8 (4.6) | 26 (11.3) | 1 (2.9) | 16 (5.9) | 19 (8.7) | | 22 (5.9) | 14 (11.0) |
|  | Strongly Disagree | 6 (3.4) | 6 (2.5) | 4 (2.0) | 4 (2.3) | 5 (2.2) | 3 (8.8) | 7 (2.6) | 6 (2.8) | | 7 (1.9) | 2 (1.6) |
| I would choose a competent veterinary technician/veterinary nurse whose demographics more closely matches mine than a veterinarian with whom there is a significant mismatch.  (n = 1024) ^b^ | Strongly Agree | 41 (22.9) | 47 (19.6) | 45 (22.5) | 43 (24.7) | 43 (18.6) | 7 (20.6) | 53 (19.6) | 51 (23.5) | | 83 (22.1) | 25 (19.7) |
|  | Agree | 51 (28.5) | 84 (35.0) | 73 (36.5) | 68 (39.1) | 81 (35.1) | 9 (26.5) | 89 (32.8) | 69 (31.8) | | 152 (40.5) | 38 (29.9) |
|  | Neutral | 72 (40.2) | 81 (33.8) | 52 (26.0) | 45 (25.9) | 58 (25.1) | 15 (44.1) | 96 (35.4) | 69 (31.8) | | 92 (24.5) | 36 (28.3) |
|  | Disagree | 8 (4.5) | 22 (9.2) | 18 (9.0) | 11 (6.3) | 30 (13.0) | 2 (5.9) | 20 (7.4) | 16 (7.4) | | 36 (9.6) | 15 (11.8) |
|  | Strongly Disagree | 7 (3.9) | 6 (2.5) | 12 (6.0) | 7 (4.0) | 19 (8.2) | 1 (2.9) | 13 (4.8) | 12 (5.5) | | 12 (3.2) | 13 (10.2) |
| I would choose a competent mid-tier veterinary professional whose demographics more closely matches mine than a veterinarian with whom there is a significant mismatch.  (n = 1026) ^b^ | Strongly Agree | 38 (21.2) | 52 (21.6) | 40 (20.0) | 41 (23.4) | 40 (17.3) | 7 (20.6) | 59 (21.7) | 53 (24.3) | | 76 (20.3) | 16 (12.6) |
|  | Agree | 59 (33.0) | 90 (37.3) | 70 (35.0) | 72 (41.1) | 81 (35.1) | 10 (29.4) | 97 (35.7) | 67 (30.7) | | 160 (42.7) | 38 (29.9) |
|  | Neutral | 71 (39.7) | 75 (31.1) | 61 (30.5) | 43 (24.6) | 64 (27.7) | 14 (41.2) | 87 (32.0) | 67 (30.7) | | 96 (25.6) | 50 (39.4) |
|  | Disagree | 4 (2.2) | 16 (6.6) | 23 (11.5) | 8 (4.6) | 35 (15.2) | 2 (5.9) | 16 (5.9) | 18 (8.3) | | 35 (9.3) | 15 (11.8) |
|  | Strongly Disagree | 7 (3.9) | 8 (3.3) | 6 (3.0) | 11 (6.3) | 11 (4.8) | 1 (2.9) | 13 (4.8) | 13 (6.0) | | 8 (2.1) | 8 (6.3) |
| I would choose a competent veterinary technician/veterinary nurse whose cultural sensitivity is higher than the veterinarian I currently see.  (n = 1024) ^b^ | Strongly Agree | 39 (21.9) | 51 (21.3) | 37 (18.5) | 61 (34.9) | 41 (17.7) | 12 (35.3) | 56 (20.7) | 52 (23.9) | | 88 (23.5) | 21 (16.5) |
|  | Agree | 52 (29.2) | 84 (35.0) | 76 (38.0) | 59 (33.7) | 72 (31.2) | 6 (17.6) | 90 (33.3) | 72 (33.0) | | 134 (35.7) | 41 (32.3) |
|  | Neutral | 70 (39.3) | 77 (32.1) | 57 (28.5) | 38 (21.7) | 67 (29.0) | 12 (35.3) | 92 (34.1) | 69 (31.7) | | 97 (25.9) | 39 (30.7) |
|  | Disagree | 7 (3.9) | 19 (7.9) | 22 (11.0) | 9 (5.1) | 34 (14.7) | 2 (5.9) | 16 (5.9) | 18 (8.3) | | 38 (10.1) | 17 (13.4) |
|  | Strongly Disagree | 10 (5.6) | 9 (3.8) | 8 (4.0) | 8 (4.6) | 17 (7.4) | 2 (5.9) | 16 (5.9) | 7 (3.2) | | 18 (4.8) | 9 (7.1) |
| I would choose a competent mid-tier veterinary professional whose cultural sensitivity is higher than the veterinarian I currently see.  (n = 1025) ^b^ | Strongly Agree | 35 (19.6) | 50 (20.8) | 40 (20.0) | 51 (29.1) | 50 (21.6) | 10 (29.4) | 55 (20.3) | 42 (19.3) | | 93 (24.8) | 26 (20.5) |
|  | Agree | 52 (29.1) | 91 (37.9) | 67 (33.5) | 71 (40.6) | 76 (32.9) | 9 (26.5) | 97 (35.8) | 73 (33.5) | | 135 (36.0) | 43 (33.9) |
|  | Neutral | 73 (40.8) | 78 (32.5) | 64 (32.0) | 44 (25.1) | 63 (27.3) | 12 (35.3) | 89 (32.8) | 76 (34.9) | | 109 (29.1) | 36 (28.3) |
|  | Disagree | 7 (3.9) | 13 (5.4) | 22 (11.0) | 5 (2.9) | 26 (11.3) | 2 (5.9) | 14 (5.2) | 16 (7.3) | | 28 (7.5) | 13 (10.2) |
|  | Strongly Disagree | 12 (6.7) | 8 (3.3) | 7 (3.5) | 4 (2.3) | 16 (6.9) | 1 (2.9) | 16 (5.9) | 11 (5.0) | | 10 (2.7) | 9 (7.1) |
| In circumstances where I experience barriers to accessing a veterinarian, I would be willing to see a veterinary technician/veterinary nurse (n = 210)^c^ | Strongly Agree | 3 (15.8) | 9 (18.4) | 11 (25.0) | 16 (37.2) | 12 (21.8) | 4 (44.4) | 7 (18.4) | 7 (15.9) | | 23 (25.6) | 10 (34.5) |
|  | Agree | 9 (47.4) | 19 (38.8) | 17 (38.6) | 17 (39.5) | 25 (45.5) | 3 (33.3) | 18 (47.4) | 21 (47.7) | | 38 (42.2) | 7 (24.1) |
|  | Neutral | 7 (36.8) | 13 (26.5) | 10 (22.7) | 6 (14.0) | 8 (14.5) | 2 (22.2) | 6 (15.8) | 10 (22.7) | | 18 (20.0) | 8 (27.6) |
|  | Disagree | 0 (0.0) | 8 (16.3) | 3 (6.8) | 1 (2.3) | 7 (12.7) | 0 (0.0) | 6 (15.8) | 4 (9.1) | | 7 (7.8) | 2 (6.9) |
|  | Strongly Disagree | 0 (0.0) | 0 (0.0) | 3 (6.8) | 3 (7.0) | 3 (5.5) | 0 (0.0) | 1 (2.6) | 2 (4.5) | | 4 (4.4) | 2 (6.9) |
| In circumstances where I experience barriers to accessing a veterinarian, I would be willing to see a mid-tier veterinary professional  (n = 210) ^c^ | Strongly Agree | 3 (15.8) | 13 (27.1) | 10 (22.7) | 13 (29.5) | 8 (14.5) | 3 (33.3) | 6 (16.2) | 11 (24.4) | | 19 (21.1) | 8 (27.6) |
|  | Agree | 8 (42.1) | 14 (29.2) | 21 (47.7) | 16 (36.4) | 24 (43.6) | 4 (44.4) | 17 (45.9) | 11 (24.4) | | 40 (44.4) | 11 (37.9) |
|  | Neutral | 6 (31.6) | 17 (35.4) | 8 (18.2) | 12 (27.3) | 16 (29.1) | 1 (11.1) | 9 (24.3) | 20 (44.4) | | 20 (22.2) | 9 (31.0) |
|  | Disagree | 2 (10.5) | 3 (6.3) | 2 (4.5) | 0 (0.0) | 5 (9.1) | 1 (11.1) | 3 (8.1) | 2 (4.4) | | 6 (6.7) | 0 (0.0) |
|  | Strongly Disagree | 0 (0.0) | 1 (2.1) | 3 (6.8) | 3 (6.8) | 2 (3.6) | 0 (0.0) | 2 (5.4) | 1 (2.2) | | 5 (5.6) | 1 (3.4) |
| In circumstances where I experience barriers to accessing a veterinarian, I would prefer to see a veterinary technician/veterinary nurse rather than forego care.  (n = 209) ^c^ | Strongly Agree | 2 (10.5) | 11 (22.9) | 14 (31.8) | 12 (27.9) | 13 (23.6) | 3 (33.3) | 9 (24.3) | 11 (25.0) | | 19 (21.1) | 10 (34.5) |
|  | Agree | 8 (42.1) | 19 (39.6) | 14 (31.8) | 20 (46.5) | 25 (45.5) | 4 (44.4) | 12 (32.4) | 13 (29.5) | | 45 (50.0) | 12 (41.4) |
|  | Neutral | 8 (42.1) | 13 (27.1) | 10 (22.7) | 7 (16.3) | 12 (21.8) | 2 (22.2) | 12 (32.4) | 15 (34.1) | | 16 (17.8) | 5 (17.2) |
|  | Disagree | 1 (5.3) | 5 (10.4) | 3 (6.8) | 2 (4.7) | 3 (5.5) | 0 (0.0) | 3 (8.1) | 4 (9.1) | | 6 (6.7) | 1 (3.4) |
|  | Strongly Disagree | 0 (0.0) | 0 (0.0) | 3 (6.8) | 2 (4.7) | 2 (3.6) | 0 (0.0) | 1 (2.7) | 1 (2.3) | | 4 (4.4) | 1 (3.4) |
| In circumstances where I experience barriers to accessing a veterinarian, I would prefer to see a mid-tier veterinary professional rather than forego care.  (n = 209) ^c^ | Strongly Agree | 2 (10.5) | 8 (16.7) | 12 (27.3) | 10 (23.3) | 16 (29.1) | 5 (55.6) | 8 (21.6) | 7 (15.9) | | 18 (20.0) | 10 (34.5) |
|  | Agree | 9 (47.4) | 17 (35.4) | 19 (43.2) | 13 (30.2) | 22 (40.0) | 3 (33.3) | 12 (32.4) | 16 (36.4) | | 37 (41.1) | 12 (41.4) |
|  | Neutral | 6 (31.6) | 18 (37.5) | 9 (20.5) | 10 (23.3) | 10 (18.2) | 1 (11.1) | 12 (32.4) | 15 (34.1) | | 20 (22.2) | 5 (17.2) |
|  | Disagree | 2 (10.5) | 5 (10.4) | 3 (6.8) | 7 (16.3) | 6 (10.9) | 0 (0.0) | 5 (13.5) | 5 (11.4) | | 12 (13.3) | 1 (3.4) |
|  | Strongly Disagree | 0 (0.0) | 0 (0.0) | 1 (2.3) | 3 (7.0) | 1 (1.8) | 0 (0.0) | 0 (0.0) | 1 (2.3) | | 3 (3.3) | 1 (3.4) |

^a Indicates that these questions were presented to the subsample of 236 participants who identified as the primary care-seeker in their household for veterinary care.^

^b Indicates that these questions were presented to the subsample of 1026 participants who reported current, recent, or future pet ownership.^

^c Indicated that these questions were presented to the subsample of 210 primary care-seekers for veterinary care and reported experiencing at least one barrier to accessing veterinary care.^

^The number of participants in each subsample who responded to each statement is shown in the table.^

**Table S6.** Cross-tabulations between participants’ perceived access to veterinary care and their perceptions of alternative veterinary service providers without collapsed categories.

|  |  | I believe a well-trained veterinary technician/veterinary nurse can provide just as high-quality service as a veterinarian (n = 232) ^a^ | | | | |
| --- | --- | --- | --- | --- | --- | --- |
|  |  | Strongly Agree  (n, %) | Agree  (n, %) | Neutral  (n, %) | Disagree  (n, %) | Strongly Disagree  (n, %) |
| In the area in which I live, it is easy for me to access veterinary care | Strongly Agree | 26 (55.3) | 31 (33.3) | 16 (30.8) | 10 (33.3) | 4 (40.0) |
|  | Agree | 12 (25.5) | 50 (53.8) | 19 (36.5) | 13 (43.3) | 2 (20.0) |
|  | Neutral | 5 (10.6) | 5 (5.4) | 13 (25.0) | 3 (10.0) | 1 (10.0) |
|  | Disagree | 2 (4.3) | 6 (6.5) | 2 (3.8) | 2 (6.7) | 1 (10.0) |
|  | Strongly Disagree | 2 (4.3) | 1 (1.1) | 2 (3.8) | 2 (6.7) | 2 (20.0) |
|  |  | I believe a well-trained mid-tier veterinary professional can provide just as high-quality service as a veterinarian. (n = 232) ^a^ | | | | |
|  |  | Strongly Agree  (n, %) | Agree  (n, %) | Neutral  (n, %) | Disagree  (n, %) | Strongly Disagree  (n, %) |
| In the area in which I live, it is easy for me to access veterinary care | Strongly Agree | 22 (44.0) | 33 (37.5) | 23 (34.8) | 6 (27.3) | 3 (50.0) |
|  | Agree | 19 (38.0) | 42 (47.7) | 24 (36.4) | 10 (45.5) | 1 (16.7) |
|  | Neutral | 2 (4.0) | 10 (11.4) | 11 (16.7) | 3 (13.6) | 1 (16.7) |
|  | Disagree | 4 (8.0) | 2 (2.3) | 5 (7.6) | 2 (9.1) | 0 (0.0) |
|  | Strongly Disagree | 3 (6.0) | 1 (1.1) | 3 (4.5) | 1 (4.5) | 1 (16.7) |
|  |  | I would choose a competent veterinary technician/veterinary nurse whose demographics more closely matches mine than a veterinarian with whom there is a significant mismatch. (n = 232) ^a^ | | | | |
|  |  | Strongly Agree  (n, %) | Agree  (n, %) | Neutral  (n, %) | Disagree  (n, %) | Strongly Disagree  (n, %) |
| In the area in which I live, it is easy for me to access veterinary care | Strongly Agree | 19 (52.8) | 21 (32.3) | 28 (32.9) | 11 (36.7) | 8 (50.0) |
|  | Agree | 11 (30.6) | 34 (52.3) | 34 (40.0) | 14 (46.7) | 3 (18.8) |
|  | Neutral | 3 (8.3) | 6 (9.2) | 14 (16.5) | 3 (10.0) | 1 (6.3) |
|  | Disagree | 1 (2.8) | 3 (4.6) | 9 (10.6) | 0 (0.0) | 0 (0.0) |
|  | Strongly Disagree | 2 (5.6) | 1 (1.5) | 0 (0.0) | 2 (6.7) | 4 (25.0) |
|  |  | I would choose a competent mid-tier veterinary professional whose demographics more closely matches mine than a veterinarian with whom there is a significant mismatch.  (n = 233) ^a^ | | | | |
|  |  | Strongly Agree  (n, %) | Agree  (n, %) | Neutral  (n, %) | Disagree  (n, %) | Strongly Disagree  (n, %) |
| In the area in which I live, it is easy for me to access veterinary care | Strongly Agree | 14 (46.7) | 24 (34.3) | 32 (36.4) | 12 (41.4) | 5 (31.3) |
|  | Agree | 12 (40.0) | 32 (45.7) | 37 (42.0) | 12 (41.4) | 3 (18.8) |
|  | Neutral | 1 (3.3) | 9 (12.9) | 13 (14.8) | 2 (6.9) | 3 (18.8) |
|  | Disagree | 0 (0.0) | 4 (5.7) | 6 (6.8) | 2 (6.9) | 1 (6.3) |
|  | Strongly Disagree | 3 (10.0) | 1 (1.4) | 0 (0.0) | 1 (3.4) | 4 (25.0) |
|  |  | I would choose a competent veterinary technician/veterinary nurse whose cultural sensitivity is higher than the veterinarian I currently see. (n = 232) ^a^ | | | | |
|  |  | Strongly Agree  (n, %) | Agree  (n, %) | Neutral  (n, %) | Disagree  (n, %) | Strongly Disagree  (n, %) |
| In the area in which I live, it is easy for me to access veterinary care | Strongly Agree | 18 (54.5) | 18 (27.7) | 33 (38.8) | 9 (27.3) | 9 (56.3) |
|  | Agree | 9 (27.3) | 36 (55.4) | 33 (38.8) | 15 (45.5) | 3 (18.8) |
|  | Neutral | 2 (6.1) | 7 (10.8) | 13 (15.3) | 4 (12.1) | 1 (6.3) |
|  | Disagree | 1 (3.0) | 2 (3.1) | 6 (7.1) | 4 (12.1) | 0 (0.0) |
|  | Strongly Disagree | 3 (9.1) | 2 (3.1) | 0 (0.0) | 1 (3.0) | 3 (18.8) |
|  |  | I would choose a competent mid-tier veterinary professional whose cultural sensitivity is higher than the veterinarian I currently see. (n = 232) ^a^ | | | | |
|  |  | Strongly Agree  (n, %) | Agree  (n, %) | Neutral  (n, %) | Disagree  (n, %) | Strongly Disagree  (n, %) |
| In the area in which I live, it is easy for me to access veterinary care | Strongly Agree | 18 (54.5) | 20 (34.5) | 36 (36.4) | 5 (19.2) | 8 (50.0) |
|  | Agree | 9 (27.3) | 29 (50.0) | 40 (40.4) | 15 (57.7) | 3 (18.8) |
|  | Neutral | 3 (9.1) | 7 (12.1) | 13 (13.1) | 3 (11.5) | 1 (6.3) |
|  | Disagree | 0 (0.0) | 1 (1.7) | 9 (9.1) | 2 (7.7) | 1 (6.3) |
|  | Strongly Disagree | 3 (9.1) | 1 (1.7) | 1 (1.0) | 1 (3.8) | 3 (18.8) |
|  |  | In circumstances where I experience barriers to accessing a veterinarian, I would be willing to see a veterinary technician/veterinary nurse. (n = 208)^b^ | | | | |
|  |  | Strongly Agree  (n, %) | Agree  (n, %) | Neutral  (n, %) | Disagree  (n, %) | Strongly Disagree  (n, %) |
| In the area in which I live, it is easy for me to access veterinary care | Strongly Agree | 24 (48.0) | 29 (33.7) | 11 (25.0) | 5 (26.3) | 4 (44.4) |
|  | Agree | 16 (32.0) | 45 (52.3) | 16 (36.4) | 8 (47.4) | 2 (22.2) |
|  | Neutral | 6 (12.0) | 6 (7.0) | 14 (31.8) | 2 (10.5) | 0 (0.0) |
|  | Disagree | 0 (0.0) | 6 (7.0) | 2 (4.5) | 3 (15.8) | 1 (11.1) |
|  | Strongly Disagree | 4 (8.0) | 0 (0.0) | 1 (2.3) | 0 (0.0) | 2 (22.2) |
|  |  | In circumstances where I experience barriers to accessing a veterinarian, I would be willing to see a mid-tier veterinary professional. (n = 208) ^b^ | | | | |
|  |  | Strongly Agree  (n, %) | Agree  (n, %) | Neutral  (n, %) | Disagree  (n, %) | Strongly Disagree  (n, %) |
| In the area in which I live, it is easy for me to access veterinary care | Strongly Agree | 26 (56.5) | 29 (34.9) | 12 (20.7) | 3 (25.0) | 3 (33.3) |
|  | Agree | 14 (30.4) | 42 (50.6) | 25 (43.1) | 7 (58.3) | 1 (11.1) |
|  | Neutral | 4 (8.7) | 5 (6.0) | 15 (25.9) | 2 (16.7) | 1 (11.1) |
|  | Disagree | 0 (0.0) | 6 (7.2) | 5 (8.6) | 0 (0.0) | 1 (11.1) |
|  | Strongly Disagree | 2 (4.3) | 1 (1.2) | 1 (1.7) | 0 (0.0) | 3 (33.3) |
|  |  | In circumstances where I experience barriers to accessing a veterinarian, I would prefer to see a veterinary technician/veterinary nurse rather than forego care. (n = 207) ^b^ | | | | |
|  |  | Strongly Agree  (n, %) | Agree  (n, %) | Neutral  (n, %) | Disagree  (n, %) | Strongly Disagree  (n, %) |
| In the area in which I live, it is easy for me to access veterinary care | Strongly Agree | 28 (54.9) | 28 (32.6) | 13 (26.5) | 2 (14.3) | 2 (28.6) |
|  | Agree | 16 (31.4) | 43 (50.0) | 20 (40.8) | 8 (57.1) | 1 (14.3) |
|  | Neutral | 5 (9.8) | 6 (7.0) | 13 (26.5) | 3 (21.4) | 0 (0.0) |
|  | Disagree | 0 (0.0) | 7 (8.1) | 3 (6.1) | 1 (7.1) | 1 (14.3) |
|  | Strongly Disagree | 2 (3.9) | 2 (2.3) | 0 (0.0) | 0 (0.0) | 3 (42.9) |
|  |  | In circumstances where I experience barriers to accessing a veterinarian, I would prefer to see a mid-tier veterinary professional rather than forego care. (n = 207) ^b^ | | | | |
|  |  | Strongly Agree  (n, %) | Agree  (n, %) | Neutral  (n, %) | Disagree  (n, %) | Strongly Disagree  (n, %) |
| In the area in which I live, it is easy for me to access veterinary care | Strongly Agree | 31 (66.0) | 26 (32.5) | 12 (23.1) | 3 (13.0) | 1 (20.0) |
|  | Agree | 12 (25.5) | 39 (48.8) | 23 (44.2) | 14 (60.9) | 0 (0.0) |
|  | Neutral | 2 (4.3) | 9 (11.3) | 12 (23.1) | 3 (13.0) | 1 (20.0) |
|  | Disagree | 1 (2.1) | 4 (5.0) | 5 (9.6) | 2 (8.7) | 0 (0.0) |
|  | Strongly Disagree | 1 (2.1) | 2 (2.5) | 0 (0.0) | 1 (4.3) | 3 (60.0) |

^a Indicates that these questions were presented to the subsample of 1026 participants who reported current, recent, or future pet ownership and who also reported being a primary care-seeker for veterinary care.^

^b Indicated that these questions were presented to the subsample of 210 primary care-seekers for veterinary care and reported experiencing at least one barrier to accessing veterinary care.^

^The number of participants in each subsample who responded to each statement is shown in the table.^

# Supplementary Figures

**Figure S1.** Primary care-seekers’ mean responses for self-reported levels of agreement with experiences related to seeking veterinary care. The red asterisk (*) indicates that the mean response for the statement is significantly different than [3] Neutral at the 0.05 level.


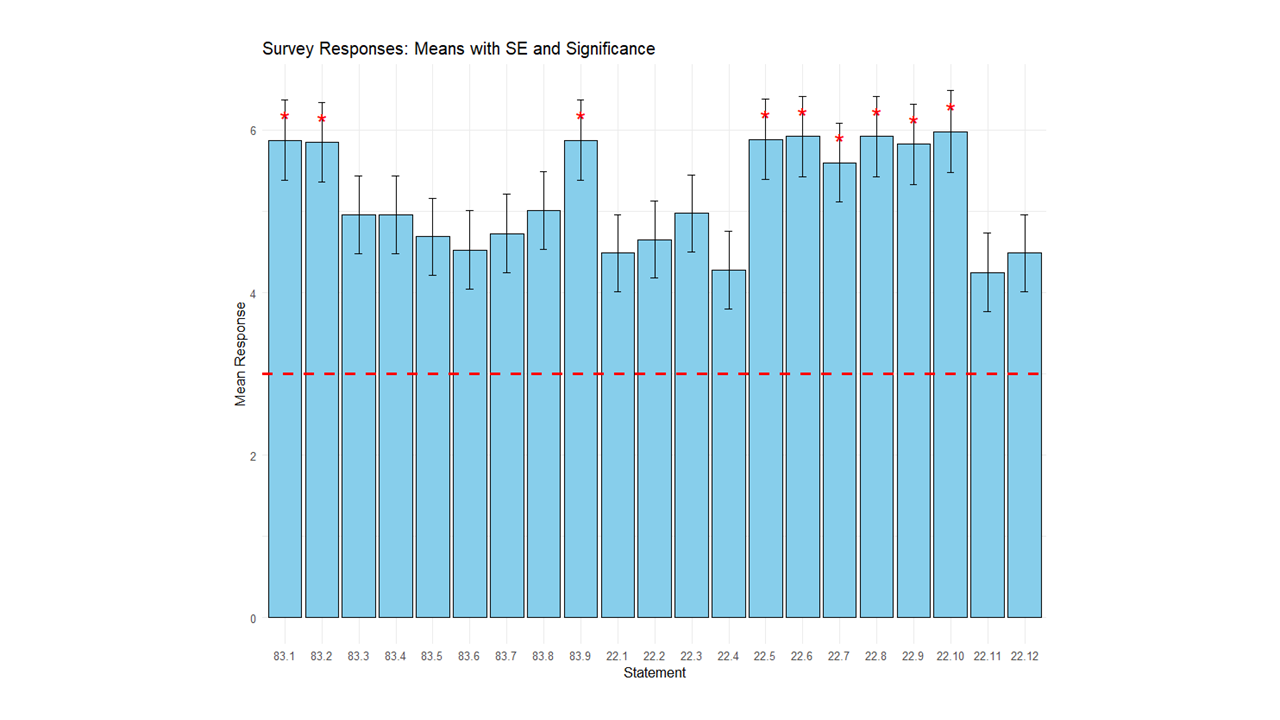
^Q83.1: In the area in which I live, it is easy for me to access veterinary care (n = 234)^

^Q83.2: I typically see my veterinarian at least once a year (n = 235)^

^Q83.3: I would prefer to have more frequent visits with my veterinarian (n = 236)^

^Q83.4: I would prefer to have less frequent visits with my veterinarian (n = 234)^

^Q83.5: It takes an unnecessarily long time to get an appointment with my veterinarian (n = 234)^

^Q83.6: My veterinary visits are too long (n = 233)^

^Q83.7: My veterinary visits are too short (n = 234)^

^Q83.8: During veterinary appointments, I wish I had more time with my veterinarian (n = 234)^

^Q83.9: My veterinarian spends an adequate amount of time with me (n = 234)^

^Q22.1: I am reluctant to seek veterinary care (n = 234)^

^Q22.2: I have had to forego seeking veterinary care in order to meet other pressing needs (e.g., my own medical care) (n = 234)^

^Q22.3: Before going to the veterinarian, I would rather search online or ask a friend for a solution (n = 234)^

^Q22.4: I have difficulty communicating with my veterinarian (n = 234)^

^Q22.5: My veterinarian makes me feel comfortable when I ask questions (n = 234)^

^Q22.6: My veterinarian answers my questions in a way I can understand (n = 234)^

^Q22.7: My veterinarian interactions with me in a way that is culturally sensitive (n = 234)^

^Q22.8: I feel respected by my veterinarian (n = 235)^

^Q22.9: I feel that my veterinarian really listens to my concerns (n = 235)^

^Q22.10: I believe my veterinarian possesses the necessary tools and expertise to treat my animal(s) (n = 235)^

^Q22.11: I am dissatisfied with the veterinarian/veterinary care options accessible to me, but they are my only choice (n = 234)^

^Q22.12: I trust my veterinarian and believe they provide the best possible care (n = 234)^

**Figure S2.** Primary care-seekers’ mean responses for self-reported frequency of satisfaction with quality of veterinary care and veterinarian-client interactions. The red asterisk (*) indicates that the mean response for the statement is significantly different than [3] Sometimes at the 0.05 level.


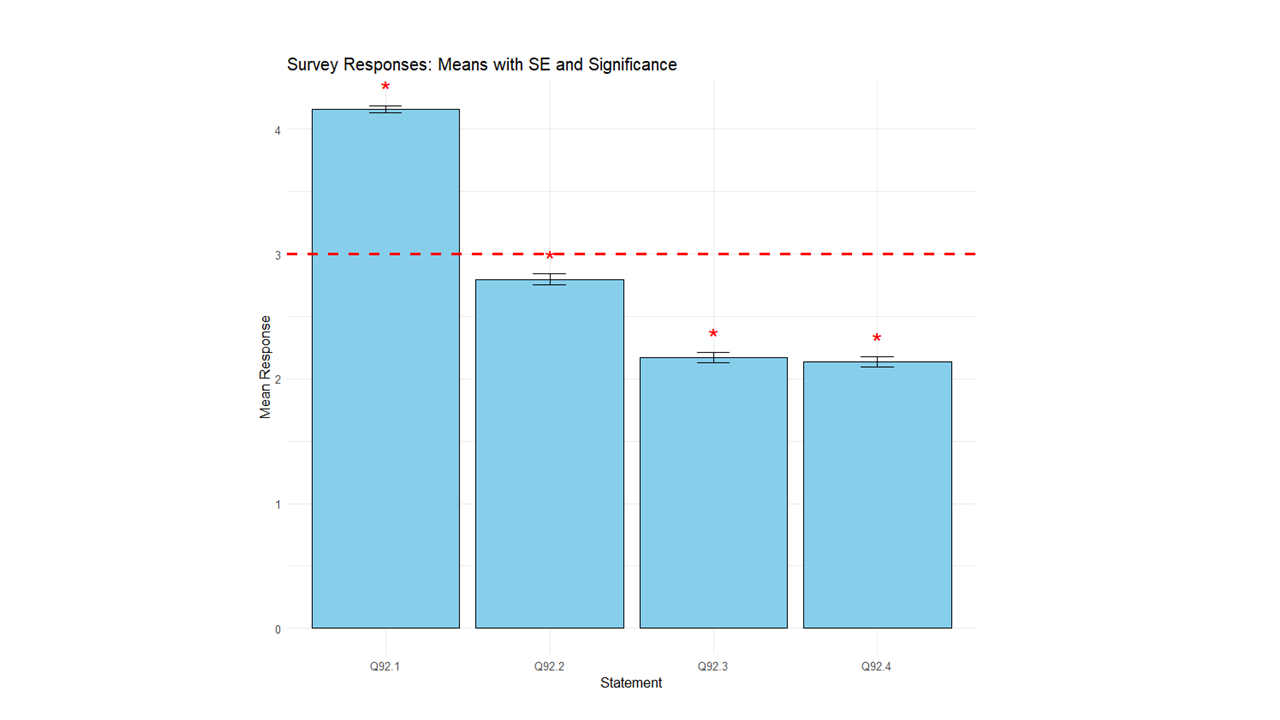


^Q92.1: I am satisfied with both the quality of care my veterinarian provides my pet and the interactions they have with me (n = 236)^

^Q92.2: I am satisfied with the quality of care my veterinarian provides my pet, but dissatisfied with the interactions they have with me (n = 236)^

^Q92.3: I am dissatisfied with the quality of care my veterinarian provides my pet, but satisfied with their interactions with me (n = 235)^

^Q92.4: I am dissatisfied with both the quality of care my veterinarian provides my pet and their interactions with me (n = 234)^

**Figure S3.** Pet owners’ mean responses for self-reported levels of agreement with perceptions of alternative veterinary service providers and willingness to seek care. The red asterisk (*) indicates that the mean response for the statement is significantly different than [3] Neutral at the 0.05 level.


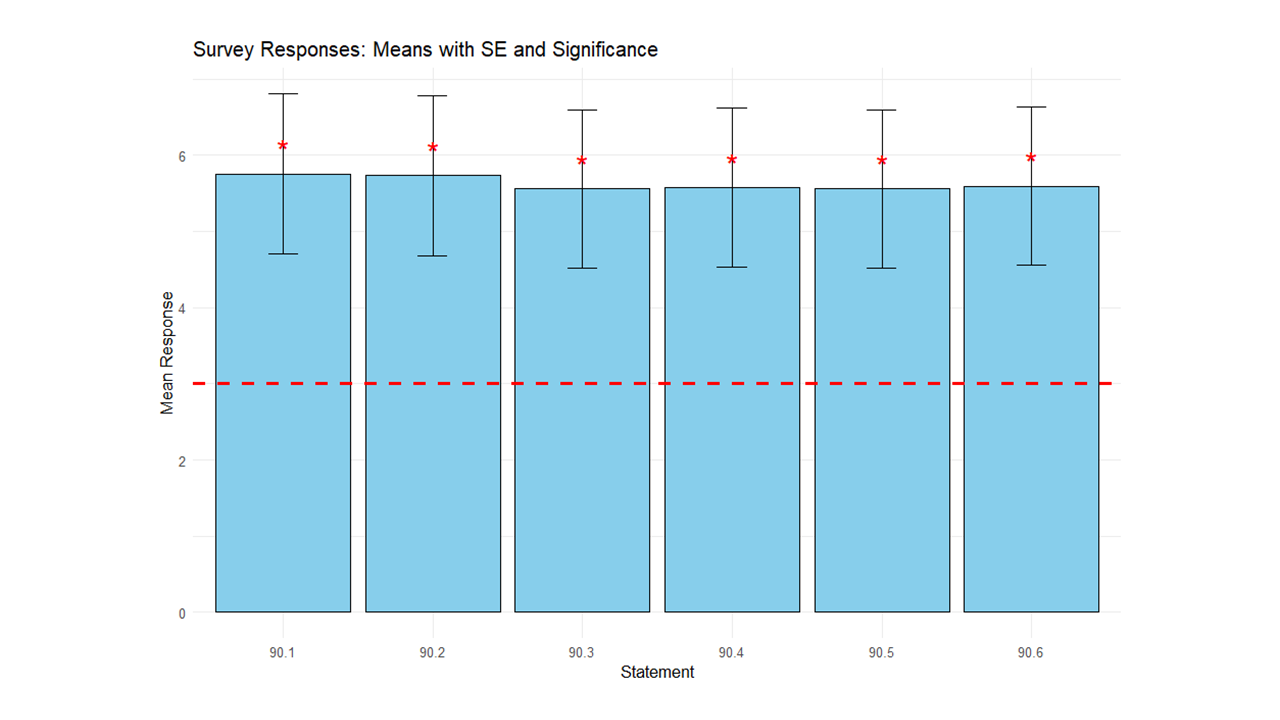


^90.1: I believe a well-trained veterinary technician/veterinary nurse can provide just as high-quality service as a veterinarian (n = 1025)^

^90.2: I believe a well-trained mid-tier veterinary professional can provide just as high-quality service as a veterinarian (n = 1024)^

^90.3: I would choose a competent veterinary technician/veterinary nurse whose demographics more closely matches mine than a veterinarian with whom there is a significant mismatch (n = 1024)^

^90.4: I would choose a competent mid-tier veterinary professional whose demographics more closely matches mine than a veterinarian with whom there is a significant mismatch (n = 1026)^

^90.5: I would choose a competent veterinary technician/veterinary nurse whose cultural sensitivity is higher than the veterinarian I currently see (n = 1024)^

^90.6: I would choose a competent mid-tier veterinary professional whose cultural sensitivity is higher than the veterinarian I currently see (n = 1025)^

**Figure S4.** Primary care-seekers’ mean responses for self-reported levels of agreement with perceptions of alternative veterinary service providers and willingness to seek care among those who reported encountering barriers to accessing veterinary care. The red asterisk (*) indicates that the mean response for the statement is significantly different than [3] Neutral at the 0.05 level.


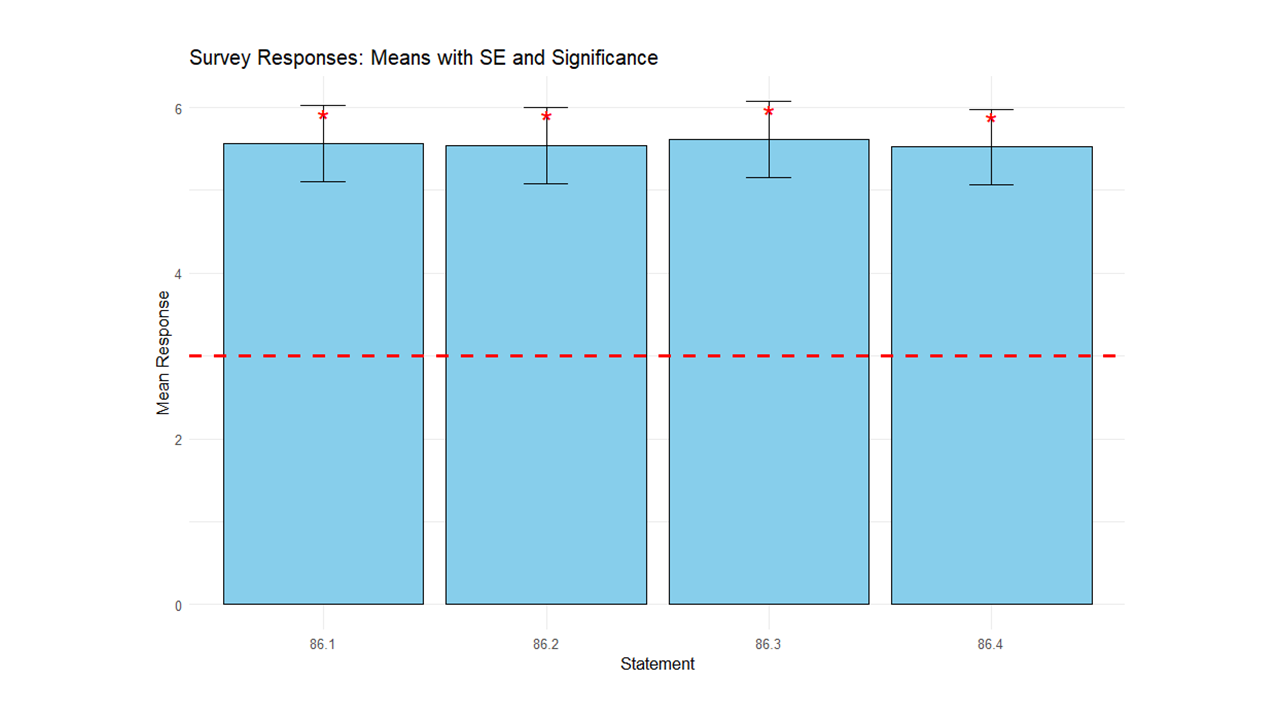


^Q86.1: In circumstances where I experience barriers to accessing a veterinarian, I would be willing to see a veterinary technician/veterinary nurse (n = 210)^

^Q86.2: In circumstances where I experience barriers to accessing a veterinarian, I would be willing to see a mid-tier veterinary professional (n = 210)^

^Q86.3: In circumstances where I experience barriers to accessing a veterinarian, I would prefer to see a veterinary technician/veterinary nurse rather than forego care (n = 209)^

^Q86.4: In circumstances where I experience barriers to accessing a veterinarian, I would prefer to see a mid-tier veterinary professional nurse rather than forego care (n = 209)^
